# Browning-induced changes in trophic functioning of planktonic food webs in temperate and boreal lakes: insights from fatty acids

**DOI:** 10.1007/s00442-022-05301-w

**Published:** 2022-12-15

**Authors:** Ursula Strandberg, Minna Hiltunen, Irena F. Creed, Michael T. Arts, Paula Kankaala

**Affiliations:** 1 Department of Environmental and Biological Sciences, University of Eastern Finland, Joensuu, Finland; 2 Department of Biological and Environmental Science, University of Jyväskylä, Jyväskylä, Finland; 3 University of Toronto-Scarborough, Toronto, Canada; 4 Department of Chemistry and Biology, Toronto Metropolitan University, Toronto, Canada

**Keywords:** Dissolved organic carbon, Eutrophication, Microbial pathway, Phytoplankton, Zooplankton

## Abstract

The effects of lake browning on trophic functioning of planktonic food webs are not fully understood. We studied the effects of browning on the response patterns of polyunsaturated fatty acids and *n*−3/*n*−6 ratio in seston and compared them between boreal and temperate lakes. We also compared the regional differences and the effects of lake browning on the reliance of zooplankton on heterotrophic microbial pathways and the mass fractions of eicosapentaenoic acid (EPA) and docosahexaenoic acid (DHA) in zooplankton. Lake browning was associated with increasing phytoplankton biomass and concentrations of EPA and DHA in both temperate and boreal lakes, but the seston *n*−3/*n*−6 ratio was lower in temperate than boreal lakes, most likely due the differences in phytoplankton community composition. The browning-induced increase in phytoplankton biomass was associated with increased reliance of zooplankton on a heterotrophic microbial pathway for both cladocerans and copepods in boreal and temperate lakes. This increased reliance on the heterotrophic microbial diet was correlated with a decrease in the EPA and DHA mass fractions in temperate copepods and a decrease in the *n*−3/*n*−6 ratio in boreal cladocerans and copepods. Our results indicate that although phytoplankton responses to lake browning were similar across regions, this did not directly cascade to the next trophic level, where zooplankton responses were highly taxa- and region-specific. These results indicate that lake browning should be considered as an overarching moderator that is linked to, e.g., nutrient increases, which have more immediate consequences on trophic interactions at the phytoplankton–zooplankton interface.

**Supplementary Information:**

The online version contains supplementary material available at 10.1007/s00442-022-05301-w.

## Introduction

Northern hemisphere lakes are browning due to higher loadings of dissolved organic carbon (DOC), and associated iron (Fe) from their watersheds (Monteith et al. 2007; Couture et al. 2012; Kritzberg and Ekström 2012). Processes behind lake browning are diverse, including climate-induced increase in precipitation and runoff and vegetation cover, as well as shortened frost period, recovery from acidification, and land-use practices (Monteith et al. 2007; Lepistö et al. 2014; Vuorenmaa et al. 2006; Hall et al. 2021). Lake browning alters internal lake processes including light attenuation, thermal stratification, nutrient cycling, as well as food web structure and functioning in lakes (Monteith et al. 2007; Creed et al. 2018). While lake browning effects on planktonic food webs have been studied, what remains unknown is the uniformality of these effects across the heterogenous conditions in the boreal vs. temperate lakes of the northern hemisphere.

Lake browning and the associated increases in nutrient loading have been suggested to show a unimodal relationship with phytoplankton biomass; phytoplankton biomass increase until a specific DOC-nutrient threshold is reached after which phytoplankton biomass starts to decline due to light limitation (Kelly et al. 2018; Bergström and Karlsson 2019; Isles et al. 2021). Additionally, lake browning generally shifts phytoplankton community composition toward taxa that: (a) can better adjust their vertical position in the water column (e.g., flagellates or cyanobacteria; Lepistö and Rosenström 1998; Carey et al. 2012); (b) are adapted to low-light conditions (Oliver and Ganf 2000); and (c) are mixotrophic or efficient in scavenging nutrients bound to organic matter (Jones 1992; Trick and Kerry 1992; Bergström et al. 2003; Senar et al. 2021). These lake browning-induced changes in phytoplankton community composition may alter the production and abundance of *n*−3 and *n*−6 polyunsaturated fatty acids (*n*−3 and *n*−6 PUFA), including eicosapentaenoic acid (EPA) and docosahexaenoic acid (DHA) (Taipale et al. 2016; Strandberg et al. 2020; Senar et al. 2019). Phytoplankton fatty acid composition is most significantly affected by phylogenetic affiliation (Galloway and Winder 2015). However, other variables, such as temperature and nutrient concentrations, have also been shown to modify the fatty acid composition of algae grown in laboratory conditions (Fuschino et al. 2011; Piepho et al. 2012; Wacker et al. 2016). An increased biomass of taxa deficient in EPA and DHA, such as cyanobacteria and chlorophytes, results in a decreased production of these biomolecules (Strandberg et al. 2015a). In contrast, an increased biomass of EPA- and DHA-rich taxa, such as flagellates and diatoms, results in increased production of these biomolecules (Taipale et al. 2013). Therefore, concentrations of EPA and DHA are dependent on both phytoplankton community composition (quality) and total phytoplankton biomass (quantity) (Strandberg et al. 2020; Wauthy and Rautio 2020; Senar et al. 2021).

The phytoplankton–zooplankton interface is a crucial first step in conveying energy and nutrients, such as n-3 and n-6 PUFA, to higher trophic levels (Müller-Navarra et al. 2000; Burns et al. 2010). Most animals cannot produce these biomolecules de novo in adequate amounts, yet they need them to maintain optimal physiological and reproductive function (Arts et al. 2001). EPA and DHA have been shown to be involved in a wide range of neural, physiological, and behavioral competences in both aquatic and terrestrial animals (Pilecky et al. 2021). Most animals are largely dependent on phytoplankton-derived PUFA, although some invertebrates have been found to contain the genetic code for the enzymes required for the synthesis of *n*−3 and *n*−6 PUFA (Kabeya et al. 2018; Babaran et al. 2020). PUFA are transferred from phytoplankton to zooplankton at higher efficiencies than carbon (Gladyshev et al. 2011), leading to a general trophic enrichment of PUFA in pelagic food webs (Kainz et al. 2004; Strandberg et al. 2015b). However, browning-induced changes in phytoplankton community composition may affect consumption rates by zooplankton, for instance due to grazer avoidance/defense or toxicity of phytoplankton (Lebret et al. 2012), possibly altering the trophic transfer efficiency of PUFA (Deininger et al. 2017). Further, the enhanced loading of DOC together with nutrients (nitrogen, phosphorus) typically promotes bacterial productivity and growth efficiency, thereby increasing the contribution of the heterotrophic basal production to the overall flow of carbon and nutrients in the food web (Arvola et al. 1996; Jansson et al. 1999; Räsänen et al. 2018). Contrary to phytoplankton, bacteria are deficient in PUFA, and thus, increased bacterial production at the base of the food web may decrease the overall production and availability of EPA and DHA to higher trophic level consumers (Johansson et al. 2016; Taipale et al. 2018). The impact of the decreased production and/or impaired trophic transfer of EPA and DHA in the planktonic food web may cascade throughout the food webs (Carpenter et al. 2015; Kankaala et al. 2019). For example, the mass fractions of EPA and DHA were significantly lower in European perch (*Perca fluviatilis*) caught from high DOC lakes in comparison to perch from low DOC lakes (Strandberg et al. 2016).

Previous studies on the trophic transfer efficiencies of PUFA are inconsistent (Taipale et al. 2018; Wauthy and Rautio 2020; Lau et al. 2021; Senar et al. 2021) and a systematic comparison across ecoregions is lacking. In this study, we compared the effects of lake browning on the trophic functioning of planktonic food webs in temperate and boreal lakes, using fatty acids biomarkers. We investigated the effects of lake browning on the selected trophic indicators to identify possible common response patterns in temperate and boreal lakes. We studied the effects of lake browning on phytoplankton PUFA concentrations, the trophic transfer of EPA and DHA at the phytoplankton–zooplankton interface, and the reliance of zooplankton on the heterotrophic microbial pathway. We hypothesized that:

1. Lake browning and the concomitant increase in nutrient loading will increase phytoplankton biomass and the overall availability of PUFA (Strandberg et al. 2020; Wauthy and Rautio 2020).
2. Lake browning will increase the reliance of zooplankton on the heterotrophic microbial pathway because of increased bacterial biomass and production at the base of the food web (Cole et al. 1988; Johansson et al. 2016; Strandberg et al. 2020).
3. Despite an increase in seston PUFA pool, the greater reliance on the heterotrophic microbial pathway will be negatively related to the mass fractions of EPA and DHA in zooplankton, because these biomolecules are scarce in the heterotrophic microbial pathway (Taipale et al. 2014).

## Materials and methods

This study focuses on comparing published data from temperate and boreal lakes. A total of 39 lakes—10 boreal and 29 temperate lakes—form the basis of this study (ESM Table S1). The mean depth of boreal and temperate lakes ranged from 1.8 to 12 m and from 2.3 to 24 m, respectively. The boreal watersheds are located in coniferous forests (typically Scots pine (*Pinus sylvestris*) and Norway spruce (*Picea abies*) on mineral soil (40 to 65% of watershed areas) and peatlands (3 to 47% of watershed areas). Lake mean DOC ranged from 2.8 to 18.9 mg L^−1^, chlorophyll *a* (Chl-a) ranged from 1.3 to 9.5 μg L^−1^, total phosphorus (TP) ranged from 1.6 to 28.8 μg L^−1^, and nitrogen (TN) ranged from 169 and 629 μg L^−1^ (ESM Table S1). The temperate watersheds are located in mixed-wood forests dominated by deciduous sugar maple (*Acer saccharum*), yellow birch (*Betula alleghaniensis*), and beech (*Fagus* spp.), with smaller presences of coniferous white pine (*Pinus strobus*), balsam fir (*Abies balsamea*), and eastern hemlock (*Tsuga canadensis*). The proportion of wetlands in the temperate watersheds ranged from 0.5 to 45%. Lake mean DOC ranged from 2.6 to 9.8 mg L^−1^, Chl-*a* ranged from 1.0 to 12.5 μg L^−1^, TP ranged from 6.0 to 48.5 μg L^−1^, and TN and 200 to 640 μg L^−1^. The temperate lake data were published in Senar et al. (2019) and the boreal lake data were published in Palviainen et al. (2016) and Strandberg et al. (2016, 2020) except for the boreal zooplankton data which have not been published.

### Sampling of seston and zooplankton

Boreal lakes were sampled between August and September in 2013, and temperate lakes were sampled between August and September in 2016. Summary of the sampling and analytical methods in the two regions is presented in the electronic supplementary material (ESM Table S2).

*Water samples*: Concentrations of TP, TN, Chl-*a*, DOC, and Fe were analyzed in boreal and temperate lakes, except for the boreal Fe concentrations which were obtained from the Hertta databank (https://www.syke.fi/avointieto). Secchi depth, pH, and SUVA_254_ were also measured. We did not have direct concentration measurements or phytoplankton biomass data for the temperate lakes. Hence, we used Chl-*a* as a proxy for phytoplankton biomass for both the temperate and the boreal lakes (Boyer et al. 2009). Chl-*a* as a proxy works with the highest precision if Chl-*a*/cell does not change with taxa, nutrient status, or light regime. While using Chl-*a* as a proxy in our comparison of lakes covering a wide range of taxa, nutrient status, and light regime is not without concern, it remains a reasonable proxy (e.g., Erratt et al. 2021).

*Seston samples*: Phytoplankton was estimated from seston samples. Boreal seston samples were collected as composite samples from the uppermost 2 m (Strandberg et al. 2020). Samples were collected from three spots within the lakes, analyzed separately, and then averaged to represent the lake. Samples were taken with Limnos water sampler (2 L) and sieved through 50 µm mesh to remove zooplankton and larger particles. Note that fatty acids and Chl-*a* were analyzed from this same pre-sieved fraction. Seston were filtered on 5 µm Durapore filters, which were immediately placed in methanol. Temperate seston samples were collected as composite samples from the epilimnion, with a plankton net (mesh 60 µm) after which the samples were sieved through 80 µm to remove zooplankton. Seston samples were filtered on 0.45 µm filters, from which the cells were washed into tubes, frozen, and lyophilized (Senar et al. 2019). Seston samples from both regions were stored at −20 °C until analysis. Previous study has shown that in these lakes, phytoplankton contributes 67–87% of total seston (Strandberg et al. 2020).

*Zooplankton samples*: Boreal zooplankton were collected from vertical hauls through the entire water column with a plankton net (mesh 200 μm) from the same site as the seston samples were collected. Samples were stored at −20 °C until analysis. In the laboratory, samples were briefly thawed and sorted to the following categories and taxa: filter-feeding cladocerans, predatory cladocerans: *Bythotrephes longimanus* (Therriault et al. 2002) and *Leptodora kindtii*, cyclopoid copepods and calanoid copepods: *Eudiaptomus* spp., *Heterocope* spp. and *Limnocalanus macrurus*. Samples comprised of 20–200 individuals. Boreal lakes had 1–6 replicate samples per taxon. We used the lake-specific means of each taxon in subsequent data analyses. Temperate zooplankton were collected from vertical hauls from the thermocline to the surfaced with a plankton net (mesh 156 µm) from the same site as the seston samples were collected. Zooplankton were immediately separated into filter-feeding cladocerans and copepods; one sample for both taxa per lake (Senar et al. 2019). Zooplankton samples from both regions were lyophilized and kept frozen (−20 °C) until analyses.

### Fatty acid analyses

Lipids were extracted with chloroform–methanol (2:1 by volume) and extracted lipids were transmethylated with 1% sulfuric acid in methanol at 90 °C for 90 min (details in Senar et al. 2019; Strandberg et al. 2020, 2022). Boreal seston samples were analyzed with Shimadzu Ultra GC–MS using Agilent DB-23 column (30 m × 0.25 mm × 0.25 µm) (Strandberg et al. 2020). Boreal zooplankton samples were analyzed with Agilent 6890 N GC equipped with 5973 N mass selective detector. The column was Agilent DB-23 (30 m × 0.25 mm × 0.25 µm), and helium was used as a carrier gas with an average velocity of 34 cm s^−1^. We used splitless injection and the inlet temperature was 250 °C. The initial oven temperature was 50 °C, which was held for 1 min, after which the temperature was increased 15 °C min^−1^ to 150 °C, then 1.5 °C min^−1^ to 190 °C, and finally 2 °C min^−1^ to 210 °C, which was held for 12 min. Temperate seston and zooplankton samples were analyzed with Shimadzu GC-2010 using SP-2560 column (100 m × 0.25 mm × 0.2 µm) (Senar et al. 2019). Identification and quantification of fatty acid methyl esters were based on mass spectra and reference standard GLC-68D (Nu-Chek-Prep). Data are presented as micrograms per mg dry weight (µg mg^−1^ DW) and/or weight percentages (w%) of total fatty acids.

### Statistical analyses

We conducted a nonparametric Mann–Whitney *U* test to evaluate the differences of water chemistry parameters, i.e., the concentrations of DOC, Fe, Chl-*a*, TP and TN, SUVA_254_, Secchi depth, pH between the temperate and boreal lakes. We also correlated lake DOC and Chl-*a* concentrations and used homogeneity of slopes test to determine the similarity of slopes between the temperate and boreal lakes. Additionally, we conducted a principal component analysis of the environmental variables of the study lakes. We calculated a novel index: Chl-*a* weighed fatty acid index to account for the effects of phytoplankton biomass which was not available for both boreal and temperate lakes. The index represents total fatty acid pool in phytoplankton, considering both the abundance of specific fatty acids (expressed as weight% of total fatty acids) and the overall phytoplankton biomass, expressed as Chl-*a* concentration. Seston fatty acid profiles were weighted by the Chl-*a* concentration as follows:

$${\text{Chl-}}a\,{\text{weighed fatty acid index}} = \frac{{{\text{FA}} w\% }}{100} \times {\text{Chl-}}a\, {\text{concentration}}{.}$$

**Fig. 1:**
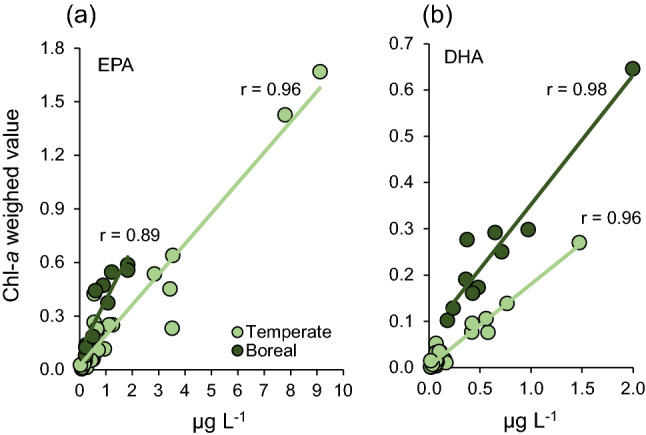
Correlations between the Chl-*a* weighed values and concentrations for **a** EPA and **b** DHA in temperate (*N* = 36) and boreal (*N* = 10) phytoplankton. Data are from Strandberg et al. (2020, 2022)

Lake-specific Chl-*a* concentration and fatty acid w% are presented in the electronic supplementary materials (ESM Table S3). Only fatty acids that are generally abundant in phytoplankton were weighted by the Chl-*a* concentration, i.e., 14:0, 16:0, 18:0, 16:1*n*−7, 18:1*n*−9, 18:1*n*−7, 16:4*n*−3, 18:3*n*−3, 18:4*n*−3, 20:5*n*−3, 22:6*n*−3, 18:2*n*−6, 18:3*n*−6, 20:4*n*−6, and 22:5*n*−6. We excluded odd-numbered and branched fatty acids, as well as ≥ C_20_ SFA from the calculation. The proportion of the excluded fatty acids accounted for 4.5–10.8% of total fatty acids in the boreal lakes and 2.9–9.6% of total fatty acid in the temperate lakes. We acknowledge that bacteria and small heterotrophic flagellates and ciliates likely contribute to the pool of saturated fatty acid (SFA), monounsaturated fatty acid (MUFA), and, to some extent, maybe even PUFA, but their overall contribution to the total fatty acid pool is much lower than that of phytoplankton (Strandberg et al. 2020). We obtained fatty acid concentration data for the boreal lakes from Strandberg et al. (2020) and for temperate lakes from Strandberg et al. (2022). We included only those cases with comparable Chl-*a* values to the temperate lakes in the current study, i.e., 1–12 µg/L, *n* = 36. The measured fatty acid concentrations (µg L^−1^) were strongly correlated with the Chl-*a* weighed indices for PUFA, MUFA and SFA in general (ESM Table S4), and EPA and DHA in specific (Fig. 1) in both regions. Thus, we conclude that the Chl-*a* weighed fatty acid indices can be used, with caution, as a proxy for fatty acid concentrations.

We performed a distance-based linear model (DistLM) to analyze the effect of environmental factors on the phytoplankton fatty acid concentrations (Chl-*a* weighed fatty acids) in boreal and temperate lake. Phytoplankton Chl-*a* weighed fatty acid data were log(*x* + 1) transformed prior to the analyses, and Euclidean distance was used as a resemblance matrix. We conducted stepwise model selection and used AIC as model fit criteria. The results are visualized with dbRDA. Continuous predictors were DOC and Fe concentration, SUVA_254_, concentrations of TP and TN, coordinates, Secchi depth, pH, lake area, and lakes are as % of watershed. Chl-*a* was not included as a predictor, because we used Chl*-a* weighed fatty acids as response variables. Predictor values were normalized prior to the analyses. The concentrations of DOC and Fe, Secchi depth and SUVA_254_ value were grouped as ‘Browning’ indicators, TP and TN concentrations were grouped as ‘Nutrient’ indicators, coordinates were grouped as ‘Region’ indicators, and lake area and lake area % of watershed were grouped as ‘Morphometry’ indicators. The browning and nutrient predictors were correlated, but we decided to keep these separate, because the slopes differed between the temperate and boreal lakes (*P* = 0.002). Furthermore, the correlation coefficients for the ‘Nutrient’ and ‘Browning’ predictors were 0.55–0.65, i.e., well below the cut-off level (0.95) for colinear predictors recommended by Anderson et al. (2008). Lake pH values were kept separate; although pH correlated with DOC concentration in the boreal lakes (*r* = −0.66), pH did not correlate with DOC concentration in the temperate lakes. Lake pH value is affected by several local characteristics and activities which may or may not be related to lake browning (including bedrock, wastewater and mining discharges, and CO_2_ concentrations). Additionally, we included ‘Sampling method’ as a categorical predictor to account for any differences due to sampling method, including seston collection and/or calculation of the Chl-*a* weighed values.

We calculated the mass fractions of major fatty acid groups in zooplankton: SFA, MUFA, and PUFA with 18–24 carbons in the acyl chain (C_18_ PUFA, C_20_ PUFA, C_22_ PUFA, and C_24_ PUFA) and bacterial fatty acids (BAFA). BAFA represent the sum of odd-chained and branched fatty acids. We used BAFA w% in zooplankton to estimate their reliance on the heterotrophic pathway. We used DistLM to analyze the explanatory power of environmental drivers on, (a) fatty acid quality indices, i.e., the content of EPA and DHA, and the *n*−3/*n*−6 in zooplankton and (b) the index for evaluating the relative importance of the heterotrophic microbial pathway for zooplankton, i.e., BAFA w% in zooplankton. We chose these distinct response factors because EPA and DHA are important nutrients for upper trophic level consumers, including fish, and impaired trophic transfer of EPA and DHA may transpire at the algae-zooplankton interface (Brett and Müller-Navarra 1997; Brett et al. 2009). Also, the *n*−3/*n*−6 ratio has been considered as an important health parameter; low *n*−3/*n*−6 ratio has been linked with, e.g., decreased cardiovascular health in humans (Kris-Etherton et al. 2002; Simopoulos 2002). In addition to the environmental predictors used in the DistLM analysis of phytoplankton fatty acids, we also included lake Chl-*a* concentration and the proportion of seston EPA, DHA and C_18_ PUFA, and heterotrophic microbial fatty acids as predictors in the model for zooplankton, i.e., ‘Seston fatty acids’. Additionally, we included categorical predictor ‘Taxa’. Taxa included in the model were: filter-feeding cladocerans, predatory cladocerans, and copepoda, excluding *Limnocalanus macrurus* which was only found in oligotrophic boreal lakes. For the boreal lakes, the calanoids *Eudiaptomus*, *Heterocope* and cyclopoids were designated as ‘copepoda’. Prior to analyses, the response variables EPA and DHA content were transformed by taking the log (*x* + 1), and the *n*−3/*n*−6 ratio and BAFA% were arcsine square-root transformed. We analyzed the Pearson correlations, using bootstrapping (1000 iterations), between the Chl-*a* concentrations and the reliance of zooplankton on the heterotrophic pathway. We also correlated the reliance of zooplankton to heterotrophic pathway with the *n*−3/*n*−6 ratio and the mass fractions of EPA and DHA in zooplankton. We present the 95% confidence interval (bias corrected) of the bootstrapped correlations. All multivariate analyses were conducted with the Primer 6 software equipped with PERMANOVA + add-on. Univariate analyses were done with IBM SPSS Statistics 27.

## Results

### Lake water chemistry and watershed characteristics

**Fig. 2:**
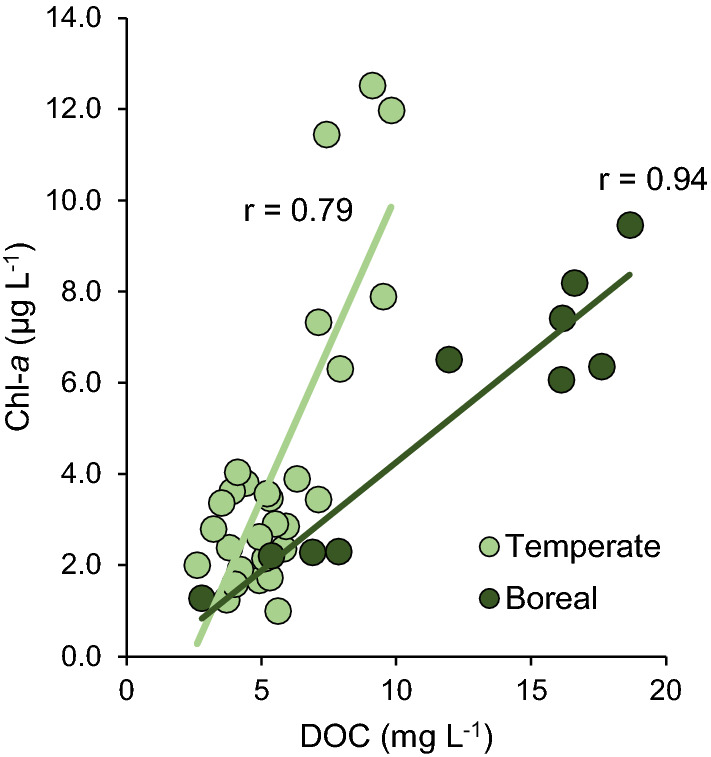
Correlations between the concentrations of DOC and Chl-*a* in the temperate (*N* = 29) and boreal (*N* = 10) lakes

Lake characteristics and detailed lake-specific physical and chemical parameters are presented in electronic supplementary material (ESM Table S1, ESM Fig. S1). The DOC concentration was higher in boreal lakes than in temperate lakes (Mann–Whitney *U* test, *p* = 0.002). Also, the SUVA_254_ values were higher in the boreal than in the temperate lakes (Mann–Whitney *U* test, *p* = 0.023), indicating differences in the quality and molecular composition of dissolved organic matter (ESM Table S1). Ranges for Secchi depth, pH, and concentrations of Chl-*a*, TP, and TN did not differ between boreal and temperate lakes. Chl-*a* increased with increasing DOC concentrations in the studied lakes, but the slope was steeper (*p* < 0.001) in the temperate than in the boreal lakes (Fig. 2).

### Phytoplankton biomass and fatty acids

**Fig. 3:**
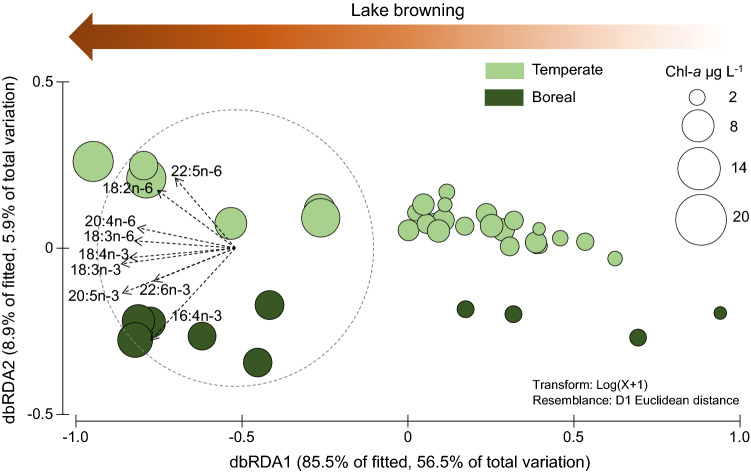
A dbRDA plot based on the DistLM model of Chl-*a* weighed fatty acids in phytoplankton (a proxy for seston fatty acid concentrations) (*N* = 39). Data were log-transformed prior to analysis. The size of the symbol represents the Chl-*a* concentration (a proxy for phytoplankton biomass) in the lakes. Note that the Chl-*a* concentration was not used as predictor in the model. Axis dbRDA1 correlated the best with lake browning indicators (Secchi depth, DOC and Fe concentrations, SUVA254). Axis dbRDA2 correlated with lake location parameters (coordinates), demonstrating regional differences. The vectors for different *n*−3 and *n*−6 PUFA, namely 16:4*n*−3, 18:3*n*−3, 18:4*n*−3, 20:5*n*−3, 22:6*n*−3, 18:2*n*−6, 18:3*n*−6, 20:4*n*−6, and 22:5*n*−6, are also shown, with the length and direction of the vector indicating the strength of correlation with the axes

The best distance-based linear model of the Chl-*a* weighed fatty acids was obtained with ‘Browning’ and ‘Location’ indicators that explained 66% of the variation in the Chl-*a* weighed fatty acids (Table 1). Model results are visualized by a dbRDA plot (Fig. 3). Lake browning indicators (DOC and Fe concentrations, SUVA_254_, and Secchi depth) explained about 57% of the variation in the phytoplankton fatty acids in the study lakes. Location, i.e., sampling coordinates, was a significant predictor in the model; after the effects of lake browning were considered, the explanatory power of location was about 9%. In both regions, the Chl-*a* weighed PUFA concentrations increased with increasing lake browning. Although the total concentration of fatty acids was driven by browning indicators, the relative contributions of n-3 and n-6 PUFA were correlated with dbRDA 2 and was driven more by ‘location’ than by ‘browning’. In general, temperate lakes were more enriched with n-6 PUFA, and boreal lakes were more enriched with *n*−3 PUFA (Fig. 3), and consequently, the mean *n*−3/*n*−6 ratio was lower in temperate (2.3 ± 1.3) vs. boreal lakes (5.7 ± 0.9) (ESM Table S3). The marginal test showed that sampling method alone could explain only 7.7% of the variation in the data set, but this was not significant (*P* = 0.056). Furthermore, sampling method did not significantly improve the model. However, we do not completely rule out that sampling method may have a minor effect, because it was strongly confounded by ‘Location’, which was a significant predictor.

**Table 1:** Marginal test results and the best model (AIC = −61.238) from the distance-based linear model of phytoplankton Chl-*a* weighed fatty acids (proxy for phytoplankton fatty acid concentrations) in the temperate and boreal lakes

Marginal test						
Indicator	res. df	regr. df	SS(trace)	Pseudo-F	*P*	Prop. explained
Nutrient	35	3	5.8055	10.497	0.0001	0.37493
Browning	33	5	8.8777	11.087	0.0001	0.57335
pH	36	2	2.092	5.6236	0.0077	0.13511
Location	35	3	4.891	8.0802	0.0005	0.31588
Sampling method	36	2	1.194	3.008	0.0557	0.0771
Morphometry	35	3	0.59426	0.69844	0.5651	0.0384

Step-wise test	res. df	regr. df	SS(trace)	Pseudo-F	*P*	Prop. explained	Cumul
Browning	33	5	8.5373	13.929	0.0001	0.57335	0.57335
+ Location	31	7	1.4302	4.1481	0.0037	0.0878	0.66114

### Zooplankton fatty acids

**Fig. 4:**
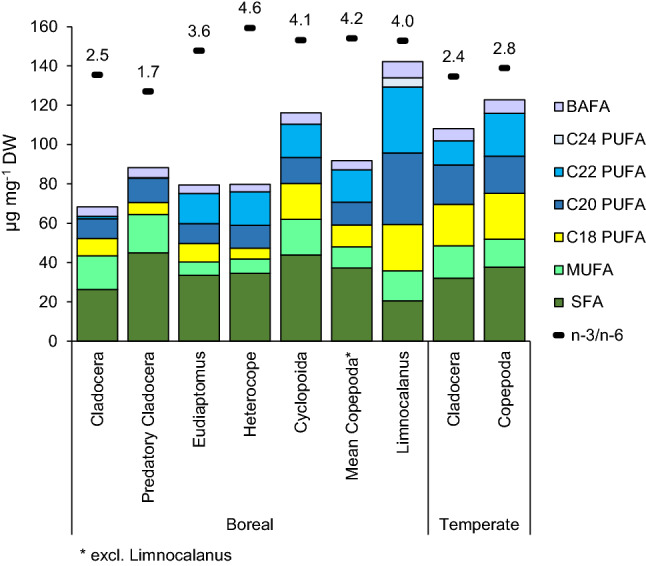
Mean fatty acid mass fractions (μg mg^−1^ DW) (*y*-axis) and the *n*−3/*n*−6 PUFA ratio (values marked in the plot) in the temperate and boreal cladocerans and copepods. In the temperate region, ‘Cladocera’ (*N* = 29) consists solely of filter-feeding taxa, and ‘Copepoda’ (*N* = 29) consists of both calanoid and cycloid copepods. In the boreal region, predatory cladocerans (*N* = 7) and filter-feeding cladocerans (*N* = 10) were presented separately, as were the cyclopoid (*N* = 4) and calanoid copepods (*Eudiaptomus* *N* = 10, *Heterocope* *N* = 7, and *Limnocalanus* *N* = 4). However, we also included a mean value for boreal copepods (cyclopoids, *Eudiaptomus* and *Heterocope*) (*N* = 21), excluding *Limnocalanus*, to facilitate the comparison to temperate copepods. Note that the C24 PUFA was only detected in the calanoid copepod *Limnocalanus*. Also note that these taxa-specific mean values represent large-scale differences between the climatic regions and not the effects of lake browning on zooplankton fatty acids

In general, cladocerans had a lower fatty acid content in boreal than in temperate lakes (Fig. 4) and the fatty acid profiles differed between groups (ESM Fig. S2). The mean proportion of PUFA in filter-feeding cladocerans was about 30% of total fatty acids for boreal lakes, and about 49% for temperate lakes (ESM Table S5). The predatory cladocerans in the boreal lakes had a higher proportion and mass fraction of SFA than the filter-feeding cladocerans (Fig. 4, ESM Table S6). Exceptionally high DHA (22:6*n*−3) proportions were found in filter-feeding cladocerans from Lake Kermajärvi, ~ 13% of all fatty acids, while the mean for across other boreal lakes was 1.9% (ESM Table S5). We repeated the analysis with additional cladoceran samples from this lake to confirm that this was not an analytical artifact. Cladocerans from the temperate lakes also contained significant amounts of DHA, on average ~ 8% of total fatty acids.

The mean proportion of PUFA in copepods was high in both boreal lakes (i.e., calanoid copepods, *Eudiaptomus* and *Heterocope*, contained ~ 42% of total fatty acids) and temperate lakes (copepods contained ~ 51% of the total fatty acids) (ESM Tables S7 and S8). The highest PUFA content, ~ 55% of total fatty acids, of all analyzed taxa, was found in the calanoid copepod *Limnocalanus macrurus* (ESM Table S9). *Limnocalanus* contained also very-long-chain PUFA (C_24_ PUFA), which were not found in any other taxa (Fig. 4). *Limnocalanus* was only found in the oligotrophic clearwater boreal lakes, and thus, the species was excluded from the analyses of lake browning-related changes in the zooplankton fatty acids and the subsequent comparisons between boreal and temperate lakes.

**Fig. 5:**
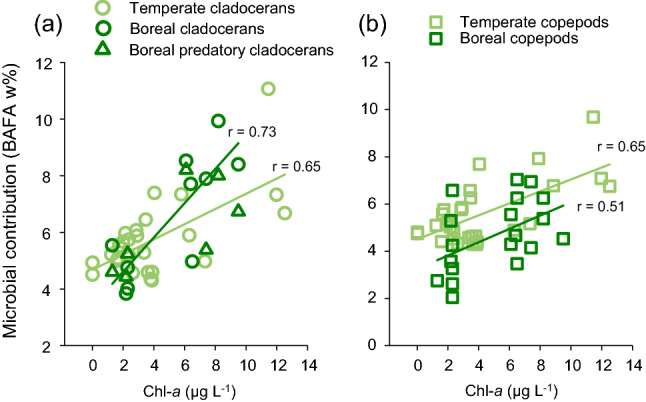
Correlations between lake Chl-*a* concentration and the microbial contribution in cladoceran and copepods (BAFA w%) in the temperate and boreal lakes. Correlations: temperate cladocerans (*N* = 29), *r* = 0.65 (*p* < 0.001, 95% confidence interval 0.11–0.86); boreal cladocerans (*N* = 17) (including both filter-feeding and predatory taxa), *r* = 0.73 (*P* < 0.001, 95% confidence interval 0.54–0.90); temperate copepods (*N* = 29), *r* = 0.65 (*P* < 0.001, 95% confidence interval 0.31–0.81); and boreal copepods (*N* = 21), *r* = 0.51 (*P* = 0.018, 95% confidence interval 0.14–0.78)

The most important factor explaining the overall reliance of zooplankton on the heterotrophic microbial pathway was phytoplankton biomass, which could explain ~ 34% of the variation in the dataset (Table 2). The model was improved by including ‘Taxa’ by 7% (*P* = 0.006) and ‘Location’ by 6% (*P* = 0.018). The best model was obtained for ‘Phytoplankton biomass’, ‘Taxa’ and ‘Location’ (*r*^2^ = 0.47). The marginal test showed that ‘Browning’ could explain 24% of the variation in the BAFA% in zooplankton, but ‘Browning’ did not improve the model after ‘Phytoplankton biomass’, ‘Taxa’, and ‘Location’ had been accounted for (Table 2). The similar relationship between Chl-*a* concentrations and the BAFA w% for cladocerans and copepods in both boreal and temperate lakes (Fig. 5) indicate that increasing reliance of zooplankton on the heterotrophic microbial pathway may be a common response to increasing phytoplankton biomass.

**Table 2:** Results from the distance-based linear model (marginal test and sequential test; best solution AIC = −676.86) of BAFA *w*% in zooplankton in the temperate and boreal lakes

Marginal test						
Indicator	Res. df	Regr. df	SS (trace)	Pseudo-F	*P*	Prop. explained
Browning	88	5	0.0242	6.8599	0.001	0.2377
Phytoplankton biomass	89	4	0.0342	15.015	0.001	0.33605
pH	91	2	0.0061	5.7776	0.018	0.05970
Seston fatty acids	88	5	0.0224	6.2193	0.001	0.22039
Taxa	90	3	0.0083	3.976	0.021	0.08118
Morphometry	90	3	0.0066	3.1239	0.057	0.06491
Location	90	3	0.0040	1.8583	0.165	0.03966

Step-wise test	Res. df	Regr. df	SS (trace)	Pseudo-F	*P*	Prop. explained	Cumul
Phytoplankton biomass	89	4	0.034	15.015	0.0001	0.33605	0.33605
+ Taxa	87	6	0.007	5.3508	0.0077	0.07273	0.40877
+ Location	85	8	0.006	4.8175	0.012	0.06019	0.46897

The environmental indicators were as follows: (1) ‘Browning’ indicator includes DOC and Fe concentration, SUVA_254_, Secchi depth; (2) ‘Phytoplankton biomass’ indicator includes concentrations of Chl-*a*, total phosphorus, and nitrogen; (3) ‘pH’; (4) ‘Seston fatty acids’ includes the proportion of EPA, DHA, C_18_ PUFA, ARA, and BAFA in seston; (5)’Taxa’ represents cladocerans, predatory cladocerans, or copepods (*Limnocalanus macrurus* excluded from the analyses); (6) ‘Morphometry’ includes lake area and lake area as % of watershed; and (7) ‘Location’ includes the coordinates representing either temperate or boreal lakes

The DistLM analysis showed that the variation in the mass fractions of EPA and DHA was largely driven by ‘Taxa’ and ‘Location’, with about 67% of the variation explained by these indicators (Table 3). Addition of ‘Morphometry’ and ‘Phytoplankton biomass’ increased the explanatory power of the model to 72%. Similarly, the variation in n-3/n-6 ratio in zooplankton was mostly explained by ‘Taxa’, about 32% of the total variation (Table 4). But contrary to the EPA and DHA mass fractions, adding ‘Seston fatty acids’ improved to model by 20% (*P* = 0.001). Other significant predictors explaining the variation in *n*−3/*n*−6 in zooplankton were: ‘Phytoplankton biomass’ and ‘Location’ (Table 4). Also, ‘Morphometry’ and ‘pH’ slightly improved the model, but the explanatory power of these indicators was only 1–2% (Table 4). ‘Browning’ was not a significant predictor for the variability of EPA and DHA mass fractions or the *n*−3/*n*−6 in zooplankton.

**Table 3:** Results from the distance-based linear model of the mass fractions of EPA and DHA in zooplankton in the temperate and boreal lakes (marginal test and sequential test; best solution had AIC = −98.347)

Marginal test						
Indicator	Res. df	Regr. df	SS (trace)	Pseudo-F	*P*	Prop. explained
Browning	88	5	27.611	9.1364	0.001	0.293
Phytoplankton biomass	89	4	5.3706	1.7957	0.127	0.057
pH	91	2	6.5583	6.8177	0.004	0.070
Seston fatty acids	88	5	8.996	2.3257	0.043	0.096
Taxa	90	3	46.217	43.438	0.001	0.491
Morphometry	90	3	3.3224	1.647	0.179	0.035
Location	90	3	24.654	15.977	0.001	0.262

Step-wise test	Res. df	Regr. df	SS (trace)	Pseudo-F	*P*	Prop. explained	Cumul
Taxa	90	3	46.217	43.438	0.001	0.491	0.491
+ Location	88	5	16.688	23.542	0.001	0.177	0.669
+ Morphometry	86	7	2.4034	3.5899	0.011	0.026	0.694
+ Phytoplankton biomass	83	10	2.7364	2.9061	0.015	0.029	0.723

The environmental indicators are described in Table 2

**Table 4:** Results from the distance-based linear model of *n*−3/*n*−6 ratio in zooplankton in the temperate and boreal lakes (marginal test and sequential test; best solution had AIC = −750.51)

Marginal test						
Indicator	Res. df	Regr. df	SS (trace)	Pseudo-F	*P*	Prop. explained
Browning	88	5	0.00345	1.0259	0.386	0.045
Phytoplankton biomass	89	4	0.00099	0.38546	0.756	0.013
pH	91	2	0.00007	0.0806	0.789	0.001
Seston fatty acids	88	5	0.01358	4.6722	0.003	0.175
Taxa	90	3	0.02462	20.949	0.001	0.318
Morphometry	90	3	0.00849	5.5366	0.007	0.110
Location	90	3	0.00741	4.7599	0.008	0.096

Step-wise test	Res. df	Regr. df	SS (trace)	Pseudo-F	*P*	Prop. explained	Cumul
Taxa	90	3	0.0246	20.949	0.001	0.318	0.318
+ Seston fatty acids	86	7	0.0153	8.7827	0.001	0.198	0.516
+ Phytoplankton biomass	83	10	0.0067	5.9624	0.001	0.086	0.601
+ Location	81	12	0.0074	12.861	0.001	0.096	0.698
+ Morphometry	79	14	0.0015	2.7156	0.085	0.019	0.717
+ pH	78	15	0.0009	3.2143	0.072	0.011	0.728

The environmental indicators are described in Table 2

**Fig. 6:**
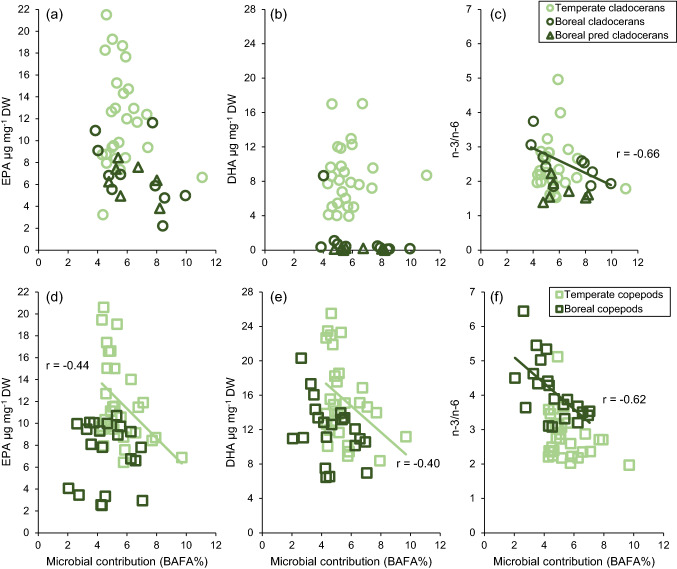
Correlations between microbial contribution in zooplankton (BAFA %) and selected PUFA indices (the concentrations of EPA and DHA, and the *n*−3/*n*−6). Panels **a**–**c** are for cladocerans, and panels **d**–**f** for copepods. Significant correlations (*P* < 0.05) shown in the figure. Sample numbers as in the Fig. 5. See Table 5 for all data

**Fig. 7:**
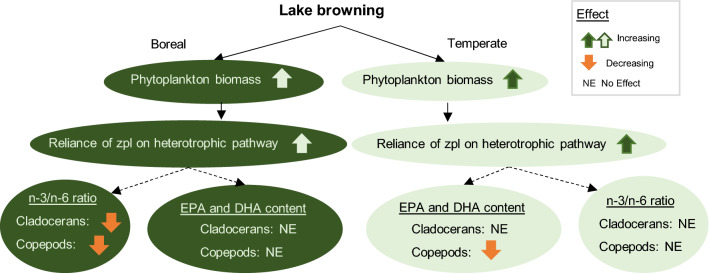
Graphical summary of the main results

‘Location’ was strongly confounded with differences in sampling methods between the two studies; thus, in models where ‘Location’ was a significant indicator, differences in the sampling methods may play a role. A strong negative correlation was observed between increasing heterotrophic microbial contribution and the *n*−3/*n*−6 ratio in boreal filter-feeding cladocerans and copepods, but not in temperate cladocerans or copepods (Table 5, Fig. 6). Instead, temperate copepods demonstrated significant negative correlation between the heterotrophic microbial contribution and the mass fractions of EPA and DHA (Table 5, Fig. 6). The main results of the study are summarized in Fig. 7.

**Table 5:** Correlation coefficients between the microbial contribution (BAFA *w*%) and the concentrations of EPA and DHA as well as the *n*−3/*n*−6 ration in temperate and boreal cladocerans and copepods

Taxa	Region	Variable	*r*	*P*	95% CI
Cladocerans	Temperate	EPA	−0.162	0.428	−0.545 to 0.503
		DHA	0.087	0.673	−0.293 to 0.509
		*n*−3/*n*−6	−0.062	0.762	−0.366 to 0.360
	Boreal	EPA	−0.516	0.127	−0.884 to 0.044
		DHA	−0.478	0.163	−0.675 to −0.511
		***n*−3/*n*−6**	**−0.663**	**0.036**	**−0.886 to −0.342**
Predatory Cladocerans	Boreal	EPA	−0.453	0.307	−0.825 to 0.267
		DHA	−0.308	0.502	−0.883 to 0.390
		*n*−3/*n*−6	−0.210	0.651	−0.957 to 0.798
Copepods	Temperate	**EPA**	**−0.442**	**0.016**	**−0.624 **to **−0.188**
		**DHA**	**−0.404**	**0.030**	**−0.607 to −0.164**
		*n*−3/*n*−6	−0.302	0.111	−0.515 to −0.038
	Boreal	EPA	0.010	0.967	−0.401 to 0.406
		DHA	−0.404	0.069	−0.701 to −0.005
		***n*−3/*n*−6**	**−0.621**	**0.003**	**−0.799 to −0.420**

Significant correlations are bolded. Correlations were done separately for filter-feeding and predatory cladocerans. Boreal copepod taxa were combined for the correlation analyses to maintain compatibility with the temperate data

## Discussion

In both regions, lake browning was linked with increased nutrient concentrations (TP, TN) and phytoplankton biomass. Lake DOC and nutrient concentrations are tightly coupled (Kortelainen et al. 2006; Palviainen et al. 2016). The range of DOC concentrations was significantly greater for the boreal than for the temperate lakes. Trends in long-term monitoring data indicate slightly greater average increase in surface water DOC concentrations for boreal regions in Europe (Monteith et al. 2007; de Wit et al. 2016) than for boreal and temperate regions in Canada (Couture et al. 2012; Hall et al. 2021). Browning of the boreal lakes in the current study was due to increased loading of DOC from coniferous forests and nutrient poor and acidic peatland (Palviainen et al. 2016). Correspondingly, pH decreased and SUVA_254_ increased along the DOC gradient in these lakes. In contrast, the watersheds of temperate lakes were mixed-wood forests dominated by deciduous forests and forested swamps and the pH and SUVA_254_ in temperate lakes did not correlate with DOC concentrations.

### Phytoplankton responses to lake browning

Lake browning increased the concentrations of nutritionally important PUFA in both the boreal and temperate lakes due to increasing phytoplankton biomass. Lake browning and the associated increase in nutrient loading have also been shown to increase phytoplankton biomass and PUFA concentration in the arctic (Wauthy and Rautio 2020). The fatty acid profiles differed between regions; the boreal lakes had systematically higher n-3/n-6 ratios than the temperate lakes. This discrepancy likely arose from differences in phytoplankton community structure (Strandberg et al. 2020; Senar et al. 2021). In the boreal study lakes, browning increased the concentration of n-3 PUFA, due to the dominance of flagellated Raphidophyte alga, *Gonyostomum semen*, which contain abundant EPA, ~ 20% of all fatty acids (Gutseit et al. 2007; Taipale et al. 2016; Strandberg et al. 2020). In the temperate study lakes, browning-induced phytoplankton biomass resulted in increased concentration of n-6 PUFA, presumably due to the dominance of cyanobacteria (Senar et al. 2021). Cyanobacteria do not contain EPA or DHA and cyanobacteria/chlorophyte dominance in mesocosms has been linked with low n-3/n-6 ratio (Strandberg et al. 2022). As noted in the results, we cannot completely exclude the confounding effect of seston sampling, which differed between the boreal and temperate lakes. Nevertheless, the estimates of phytoplankton community composition support the conclusion that the regional differences in fatty acid concentrations and *n*−3/*n*−6 ratio are predominantly caused by the differences in phytoplankton community composition (Strandberg et al. 2020; Senar et al. 2021), and not methodological differences.

Previous studies have documented changes in phytoplankton community composition with lake browning. Senar et al. (2021) observed in temperate lakes that moderate DOC concentrations promote cyanobacteria prevalence, while further DOC concentrations shift the phytoplankton community composition toward flagellates and mixotrophic taxa. In comparison to the boreal lakes, temperate lakes typically experience a longer and warmer growing season and may be more susceptible to cyanobacteria prevalence. Specifically, longer and stronger thermal stratification in summer has been noted to increase the frequency and duration of cyanobacteria blooms (Huber et al. 2012). The northern lakes have a shorter growing season, and the watersheds of the browning boreal lakes in this study consisted of more acidic nutrient poor peatland (Palviainen et al. 2016). The lower pH in browning boreal lakes may create a competitive disadvantage for cyanobacteria which generally prefer higher pH (Brock 1973; Mangan et al. 2016). Cyanobacteria blooms have not been a major concern in these browning boreal lakes; however, another nuisance alga, *Gonyostomum semen*, has been increasingly causing problems across northern Europe (Lepistö et al. 1994; Trigal et al. 2013; Johansson et al. 2016). *Gonyostomum semen* seems to favor small lakes that have brown watercolor, low pH, and high concentrations of DOC and phosphorus (Lepistö et al. 1994; Trigal et al. 2013).

### Zooplankton responses to lake browning

The importance of the heterotrophic microbial pathway at the producer–consumer interface increased with lake browning in both regions. However, zooplankton reliance on the heterotrophic microbial diet responded more strongly to the increasing phytoplankton biomass than DOC concentration. This is concordant with previous studies, indicating that the heterotrophic microbes efficiently utilize phytoplankton exudates (Middelboe and Søndergaard 1993; Malinsky-Rushansky and Legrand 1996; Guillemette et al. 2013). Increased loading of allochthonous carbon may also fuel bacteria; however, allochthonous DOC in the boreal lakes was primarily comprised of high-molecular-weight compounds (Strandberg et al. 2020), which are less biologically available (more recalcitrant) for bacteria than phytoplankton exudates. Thus, our results suggest that in browner lakes, it is ultimately the increasing phytoplankton biomass and autochthonous DOC that drives the increased reliance of zooplankton on the heterotrophic microbial pathway.

Despite the increasing contribution of the heterotrophic microbial pathway in browning lakes, the abundance of PUFA in zooplankton indicates that the autotrophic pathway remains prevalent. It is noteworthy that the increasing heterotrophic microbial contribution did not affect the total fatty acid content in cladocerans or copepods, indicating that their nutritional status was not compromised. The DHA mass fraction was higher-than-expected in zooplankton from certain temperate and boreal lakes (Fig. 7B); similarly, higher-than-expected values has been previously found in cladocerans in some Swedish lakes (Lau et al. 2021). The phytoplankton fatty acids could not explain the DHA values in zooplankton. Cladocerans may have fed on phytoplankton patches with high DHA concentration (Schatz and McCauley 2007), or even selective feeding has been suggested (Hartman and Kunkel 1991). Cladocerans have been noted to have limited capacity to desaturate and elongate 18:3*n*−3 or 20:5*n*−3 to DHA. In fact, the C_22_ PUFA are preferentially retroconverted to C_20_ PUFA (Strandberg et al. 2014); thus, we do not think that the bioconversion of precursor fatty acids explains the higher-than-expected levels of DHA in these cladocerans.

Lake browning was not an important predictor for the *n*−3/*n*−6 ratio and the mass fractions of EPA and DHA in zooplankton. The mass fractions of EPA and DHA in zooplankton were independent from the phytoplankton fatty acids and lake browning. Similar results have also been reported from browning arctic lakes (Wauthy and Rautio 2020). ‘Taxa’ was the most important factor explaining the variation in these response variables, which is in accordance with previous studies on zooplankton fatty acids (Hiltunen et al. 2015). However, although ‘taxa’ was the most important predictor also for *n*−3/*n*−6 ratio in zooplankton, phytoplankton fatty acids could explain about 20% of the variation of *n*−3/*n*−6 in zooplankton in boreal and temperate lakes. This was mainly due to C_18_ PUFA. Although lake browning, or more specifically increased phytoplankton biomass, increased the reliance of zooplankton on the heterotrophic microbial pathway in both temperate and boreal lakes, the impact on EPA and DHA mass fractions and the n-3/n-6 ratio in zooplankton differed between the lakes. In boreal lakes, the increased reliance on the heterotrophic microbial pathway did not significantly impact the mass fractions of EPA and DHA, albeit a negative trend was observed, but significantly decreased the *n*−3/*n*−6 ratio in zooplankton. By contrast, in temperate lakes, the increasing reliance on the heterotrophic microbial pathway correlated negatively with the EPA and DHA content in copepods, but not in cladocerans. These findings indicate that although phytoplankton PUFA concentrations respond strongly to lake browning, these changes do not necessarily cascade up to the level of zooplankton. However, DOC concentration has been noted to explain 6–16% of zooplankton fatty acid composition in boreal lakes (Hiltunen et al. 2015). It is likely that ecological factors play an integral role in the overall trophic transfer of energy and important micronutrients in freshwater food webs. Factors, such as defense mechanisms and predator avoidance of phytoplankton, as well as zooplankton community composition, body size, and feeding modes (Johansson et al. 2013; Wenzel et al. 2021), strongly affect the trophic transfer efficiency of PUFA; indicating that the effects of increased DOC concentrations on zooplankton fatty acids are variable and likely system dependent.

Lake browning-related effects on zooplankton community composition may have a major impact on the overall PUFA pool in freshwater food webs. *Limnocalanus* is a glacial relict and has specific environmental requirements, including cold, well-oxygenated water; thus, this species is vulnerable to environmental change, including lake browning (Segerstråle 1976; Kane et al. 2004). It is unlikely that this species was present in the temperate lakes of this study, but it has been found in the Laurentian Great Lakes (Nasworthy et al. 2020). Because of the restricted environmental requirements, the evaluation of lake browning on the fatty acids in *Limnocalanus* is not meaningful. However, *Limnocalanus* has a very high PUFA content and is also a large-bodied copepod; thus, the absolute PUFA amount per individual is high (Hiltunen et al. 2014). This suggests that *Limnocalanus* is a key taxon for the trophic transfer of EPA and DHA to planktivorous fish in planktonic food webs of large, oligotrophic lakes (Strandberg et al. 2018, Nasworthy et al. 2020). These large-scale differences in zooplankton community composition may partly explain why browning-related decrease in EPA and DHA mass fractions in the European perch were previously reported from these same boreal lakes (Strandberg et al. 2016), even if the within-taxa values in zooplankton were not affected by lake browning indicators.

## Conclusion

Browning, or more specifically, the concurrent nutrient loading, increased phytoplankton biomass and PUFA concentrations in both the boreal and temperate lakes. Browning-induced increase in phytoplankton biomass correlated with increased reliance of zooplankton on the heterotrophic pathway in both the temperate and boreal regions. The concentrations of PUFA and the *n*−3/*n*−6 ratio in zooplankton were highly taxon- and region-specific. The taxon-specificity of zooplankton indicates that changes in the community composition may be a key mechanism affecting the total PUFA pool in primary consumers and thus trophic transfer to upper trophic level consumers. Specifically, the absence of key taxa, such as *Limnocalanus*, in boreal browning lakes may decrease EPA and DHA transfer to fish. This suggests that including the responses on zooplankton community composition may improve our understanding of the effects of lake browning and/or eutrophication on the total availability and trophic transfer of PUFA in aquatic food webs. Furthermore, the observed region-specificity suggests that large-scale generalizations of the effects of lake browning to zooplankton fatty acids may not be meaningful, as the effects are likely highly system-specific and presumably dependent on other environmental and ecological factors.

## Supplementary Information

Below is the link to the electronic supplementary material.

Supplementary file1 (PDF 13837 KB)

## Data Availability

The data are available from the corresponding author on reasonable request.

## Code availability

N/A.
