# Supplementary material for: Browning-induced changes in trophic functioning of planktonic food webs in temperate and boreal lakes: insights from fatty acids
Source: Oecologia. 2022 Dec 15;201(1):183–97. doi: 10.1007/s00442-022-05301-w (PMC9813244; doi:10.1007/s00442-022-05301-w)
Supplement: Supplementary file 1 — Supplementary file1 (PDF 13837 KB) [file 442_2022_5301_MOESM1_ESM.pdf]

Electronic Supplementary Material

Browning-induced changes in trophic functioning of planktonic food webs in temperate and boreal lakes: insights from fatty acids

Ursula Strandberg[1], Minna Hiltunen[1,2], Irena F. Creed[3], Michael T. Arts[4], Paula Kankaala[1]

[1] University of Eastern Finland, Department of Environmental and Biological Sciences, Joensuu, Finland
[2] University of Jyväskylä, Department of Biological and Environmental Science, Jyväskylä Finland
[3] University of Toronto Scarborough, Toronto, Canada
[4] Toronto Metropolitan University, Department of Chemistry and Biology, Toronto, Canada

Table S1. Location and water quality parameters of the study lakes. Water chemistry parameters include total phosphorus and nitrogen concentrations, chl *a* concentration, Secchi depth, DOC concentration, $SUVA_{254}$ and pH.

Lake	Region	Latitude	Longitude	TP	TN	Chl *a*	Secchi depth	DOC	$SUVA_{254}$	pH
				µg/L	µg/L	µg/L	m	mg/L		
Hattujärvi	Boreal	62.95234	31.1957	23	377	7.4	1.2	16.2	4.3	6.4
Harkkojärvi	Boreal	62.95527	31.0420	18	458	8.2	1.1	16.6	3.9	6.0
Koitere	Boreal	63.01760	30.7796	11	286	6.5	1.4	11.9	4.2	6.3
Nuorajärvi	Boreal	62.68494	31.1363	22	328	6.1	1.2	16.1	4.4	5.6
Mekrijärvi	Boreal	62.76636	30.9535	29	556	9.5	1.0	18.7	4.1	6.2
Ylinen	Boreal	62.59592	30.2251	4	315	2.3	4.4	6.9	3.1	7.0
Kermajärvi	Boreal	62.44841	28.6842	5	365	2.3	4.1	7.9	2.9	7.2
Ätäskö	Boreal	62.05405	29.9809	25	629	6.4	1.3	17.6	3.6	7.2
Karjalan Pyhäjärvi	Boreal	61.86335	29.9951	5	219	2.2	5.1	5.4	2.4	7.3
Kuorinka	Boreal	62.61311	29.4034	2	169	1.3	9.0	2.8	1.7	7.1
Bass	Temperate	44.67679	-78.5338	11	430	2.4	4.0	5.8	2.8	7.9
Bella	Temperate	45.44625	-79.0223	7	210	2.0	4.0	2.6	2.0	6.8
Brandy	Temperate	45.10896	-79.5223	49	640	7.9	1.0	9.5	5.0	6.8
Couchiching	Temperate	44.65381	-79.3648	13	530	3.5	3.0	5.3	1.2	8.1
Crystal	Temperate	44.75829	-78.4796	21	450	1.7	6.5	4.9	2.4	8.1
Davis	Temperate	44.79	-78.71	8	320	1.7	5.1	5.3	2.3	7.7
Depensiers	Temperate	46.31159	-79.4149	20	610	12.5	1.6	9.1	2.8	7.2
Devil's	Temperate	44.86848	-78.8335	12	250	3.9	3.8	4.2	2.7	6.5
Four Mile	Temperate	44.66894	-78.7421	7	330	1.0	5.2	5.6	1.6	8.1
Fox	Temperate	45.38855	-79.357	11	380	7.3	2.0	7.1	4.0	6.2
Head	Temperate	44.73627	-78.9049	11	410	3.8	2.6	4.4	1.7	7.9
Kashagawigamog	Temperate	44.9927	-78.593	8	280	1.9	5.5	4.2	2.6	7.5
Koshlong	Temperate	44.97	-78.4897	7	260	2.4	5.6	3.8	3.0	6.6
Loom	Temperate	44.74514	-78.4615	7	400	2.9	4.5	5.9	2.1	8.0
Loon	Temperate	45.01105	-78.3789	9	420	3.6	5.2	5.2	2.8	7.7
MacLean	Temperate	44.81714	-79.6563	19	480	11.5	1.3	7.4	2.3	7.2
Maple	Temperate	45.10045	-78.6639	8	260	1.2	5.0	3.7	2.9	7.1
Mary	Temperate	45.26181	-79.2388	12	390	2.2	3.2	5.1	3.4	6.7
Menominee	Temperate	45.19443	-79.1366	11	400	3.4	3.5	7.1	3.9	6.4
Mink	Temperate	46.18256	-79.2205	18	480	12.0	2.0	9.8	2.9	6.7
Morrison	Temperate	44.86661	-79.4516	9	330	2.9	2.7	5.5	2.9	6.8
Oxbow	Temperate	45.43925	-78.9676	7	220	1.6	4.1	4	2.7	6.6
Paint	Temperate	45.22143	-78.9452	10	260	3.6	3.0	3.9	2.5	6.9
Raven	Temperate	45.20548	-78.8514	6	200	3.4	4.2	3.5	2.6	6.3
Ril	Temperate	45.16873	-79.0053	11	300	4.0	3.4	4.1	4.8	6.6
Sparrow	Temperate	44.81015	-79.3813	13	440	2.6	3.5	4.9	2.3	7.9
Tea	Temperate	44.86992	-79.6468	8	380	3.9	3.5	6.3	2.8	7.5
Twelve Mile	Temperate	45.02005	-78.7091	8	300	2.8	4.2	3.2	2.4	7.0
Wasi	Temperate	46.14238	-79.2273	32	530	6.3	0.7	7.9	3.3	6.9

Table S2. Comparison of sampling details for temperate and boreal lakes as well as brief description of analytical methods. See Senar et al. (2019) and Strandberg et al. (2020) for the details.

	Boreal	Temperate
Number of lakes	10	29
Sampling occasion	Aug-Sep 2013	Aug-Sep 2016
Phytoplankton sampling depth	Composite of 0-2 m from the surface	Composite of epilimnion
Phytoplankton sampling	Limnos water sampler (2 L)	Plankton net mesh size 60 µm
Screening for phytoplankton	Mesh size 50 µm	Mesh size 80 µm
Seston filtering	5 µm filters. Lipids extracted from filters.	0.45 µm filters and cells washed from filters into tubes. Lyophilized.
Zooplankton sampling	Vertical hauls with plankton net (mesh size 200 µm) from near the bottom to the surface	Vertical hauls with plankton net (mesh size 156 µm) from thermocline to the surface
Zooplankton identification	Microscopic. Separation into filter-feeding cladocerans, predatory cladocerans, cyclopoids, *Eudiaptomus* sp., *Heterocope* sp. or *Limnocalanus macrurus*	Microscopic. Separation into filter-feeding cladocerans or copepods
Lipid extraction	Chloroform:methanol (2:1 by volume)	Chloroform:methanol (2:1 by volume)
Fatty acid derivatization	Acid catalyzed (1% sulfuric acid in methanol), 90 min. at 90°C	Acid catalyzed (1% sulfuric acid in methanol), 90 min. at 90°C
GC analysis and detection	Shimadzu Ultra and Agilent 6890N with MS 5973N, MS detection, column DB-23	Shimadzu GC2010 plus, FID, column SP-2560

Table S3. Mean chlorophyll *a* (μg L^{-1}) and the n-3/n-6 PUFA ratio of seston, and seston fatty acids proportion (weight % of total fatty acids) in boreal (North Karelia, Finland) and temperate (Ontario, Canada) lakes. The fatty acid data include the major fatty acids, which account for 87.5 – 98.5% of total fatty acids.

Lake	Chl-*a*	n3/n6	14:0	iso15:0	ai15:0	15:0	16:0	17:0	i17:0	18:0	20:0	22:0	24:0	16:1n-7	18:1n-9	18:1n-7	16:4n-3	18:3n-3	18:4n-3	20:5n-3	22:6n-3	18:2n-6	18:3n-6	20:4n-6	22:5n-6
Bass	2.4	2.3	7.2	1.1	0.4	1.7	29.0	1.8	0.6	7.9	0.3	0.4	0.2	7.6	6.7	2.6	0.3	7.3	4.8	4.9	3.1	4.4	0.7	1.9	1.7
Bella	2.0	2.6	5.3	1.1	0.5	1.4	20.2	0.9	0.7	8.6	0.6	0.9	0.5	9.3	7.6	3.1	0.3	8.8	4.0	6.8	4.6	4.5	0.5	2.6	1.9
Brandy	7.9	2.3	3.8	2.1	0.5	1.8	27.5	1.5	0.7	5.9	0.3	0.3	0.2	6.6	6.6	3.0	0.2	16.9	3.1	3.3	0.7	7.7	0.7	1.9	0.4
Couchiching	3.5	1.7	9.1	1.9	0.6	1.8	21.7	1.4	1.2	5.8	0.4	0.4	0.3	5.5	5.9	2.6	0.2	5.8	5.1	7.2	5.4	6.2	0.7	4.5	2.4
Crystal	1.7	2.7	6.2	1.4	0.5	1.4	24.3	1.5	1.0	7.4	0.7	0.5	0.2	4.8	5.6	2.3	0.3	8.4	5.1	6.6	5.5	4.3	0.6	2.0	2.7
Davis	1.7	1.3	5.0	1.1	0.4	0.6	26.7	1.6	1.2	9.6	1.3	1.1	0.5	9.8	5.3	4.8	0.1	3.7	2.8	3.7	2.9	4.6	0.6	2.3	2.7
Depensiers	12.5	1.4	23.2	1.7	1.6	1.4	21.0	0.8	0.6	4.8	0.2	0.3	0.3	5.2	5.0	1.4	0.3	5.8	6.2	2.4	2.4	6.6	1.0	1.3	3.4
Devil's	3.9	1.5	10.5	1.0	0.6	1.3	22.9	0.5	0.6	4.9	0.4	0.5	0.3	4.9	7.5	2.5	0.5	6.0	9.5	3.5	2.9	6.1	1.4	2.8	4.3
Four Mile	1.0	1.9	7.9	1.7	0.6	1.3	27.7	1.8	1.0	7.2	0.6	0.7	0.2	5.3	7.2	3.5	0.2	5.6	3.9	4.9	5.0	4.7	0.4	2.5	2.7
Fox	7.3	1.4	10.1	0.9	0.3	2.1	21.2	0.7	0.3	3.8	0.4	0.4	0.5	2.9	7.0	2.9	0.4	10.9	5.4	4.3	3.8	9.2	1.0	2.1	4.8
Head	3.8	2.0	10.7	0.9	0.4	1.4	21.6	1.0	0.5	5.3	0.3	0.4	1.1	6.7	7.3	1.8	0.4	7.1	6.6	5.1	5.3	5.5	0.9	2.5	3.1
Kashagawigamog	1.9	2.2	6.1	1.0	0.4	1.4	23.2	1.4	0.9	7.3	0.5	0.4	0.3	5.4	5.7	3.5	0.2	7.8	4.5	6.2	6.3	5.1	0.5	3.5	2.3
Koshlong	2.4	15.7	7.4	0.3	0.1	1.5	16.1	0.3	0.2	1.8	0.1	0.2	0.3	2.0	18.8	0.8	0.6	1.3	11.6	16.1	13.5	1.3	0.2	0.6	0.6
Loom	2.9	2.0	7.8	1.8	0.7	2.0	27.2	1.8	1.2	6.7	0.4	0.6	0.3	4.9	6.9	4.1	0.2	7.0	3.9	4.7	3.3	5.1	0.4	2.3	1.8
Loon	3.6	2.7	9.7	1.5	0.5	1.7	24.9	1.2	1.4	6.9	0.4	0.5	0.2	4.7	5.0	2.4	0.5	8.0	7.1	5.1	4.8	4.8	0.5	2.1	2.2
MacLean	11.5	1.7	8.5	2.0	0.4	2.2	28.8	1.8	1.1	8.4	0.4	0.6	0.9	3.4	8.0	1.6	0.3	7.6	3.5	2.4	2.8	7.2	0.4	1.2	1.1
Maple	1.2	2.0	7.5	1.1	0.4	1.3	22.0	1.3	0.7	8.4	0.4	0.4	0.3	5.2	7.3	3.5	0.2	7.7	5.1	5.8	5.5	5.7	0.7	3.7	2.2
Mary	2.2	2.0	5.5	1.2	0.4	1.6	27.4	1.9	1.2	11.5	0.6	0.7	0.7	5.1	5.6	2.7	0.2	6.4	3.2	4.4	4.7	4.5	0.5	2.2	2.3
Menominee	3.4	5.4	8.0	0.6	0.2	1.3	27.8	0.4	0.3	3.8	0.2	0.4	0.5	2.1	21.8	1.0	0.1	2.7	6.2	7.1	9.4	2.8	0.4	0.9	0.7
Mink	12.0	2.0	10.9	0.7	0.8	2.7	17.6	0.6	0.4	5.2	0.2	0.4	0.5	4.7	9.4	2.2	0.2	6.5	13.2	3.6	2.3	5.0	1.1	2.5	4.4
Morrison	2.9	2.2	7.3	1.0	0.4	1.6	22.0	0.9	0.5	5.2	0.4	0.6	0.7	6.3	10.3	2.3	0.2	7.0	6.0	6.8	5.4	5.1	0.9	3.0	2.3
Oxbow	1.6	2.0	12.9	1.2	0.5	2.5	15.8	1.1	0.5	6.1	0.4	0.7	0.4	4.5	6.6	2.7	0.3	8.3	9.4	5.3	3.3	5.4	0.8	3.4	3.5
Paint	3.6	1.3	21.1	1.3	1.1	2.2	22.2	0.5	0.8	4.8	0.3	0.5	0.3	4.9	7.9	1.6	0.1	3.9	5.0	3.6	2.6	5.3	0.8	2.0	3.8
Raven	3.4	1.3	14.5	1.2	0.5	2.7	16.9	0.9	0.6	6.5	0.3	0.4	0.3	3.7	6.6	2.6	0.2	5.4	5.2	6.4	3.9	5.2	0.8	5.5	5.1
Ril	4.0	1.9	8.1	0.9	0.4	2.4	21.6	0.3	0.1	8.0	0.3	0.3	0.4	4.9	6.7	2.2	0.5	8.7	11.8	3.3	2.5	7.6	1.5	1.7	3.2
Sparrow	2.6	1.8	6.1	1.1	0.2	1.3	31.9	1.4	0.7	7.3	0.4	0.5	0.3	6.2	7.6	3.4	0.4	6.4	3.9	4.3	3.0	5.4	0.6	2.2	1.8
Tea	3.9	1.4	12.6	1.0	0.4	2.0	22.5	0.5	0.2	4.0	0.2	0.2	0.2	4.4	7.9	1.9	0.2	5.9	6.8	5.2	4.2	6.2	1.7	3.1	5.2
Twelve Mile	2.8	2.0	10.3	1.2	0.6	2.5	21.5	0.6	0.4	4.6	0.3	0.4	0.6	13.5	5.7	3.7	0.3	7.6	4.7	5.4	2.3	4.7	1.0	2.5	1.9
Wasi	6.3	4.7	5.2	1.3	0.3	1.5	24.5	1.6	0.6	4.2	0.2	0.3	0.4	2.2	4.3	1.9	0.3	16.7	10.3	7.4	4.3	4.3	0.4	1.4	2.2
Kuorinka	1.3	5.1	9.4	1.1	0.6	0.9	21.9	0.5	0.3	6.0	0.6	1.1	1.7	5.0	4.6	1.2	0.8	6.2	7.2	6.3	13.3	2.8	0.8	1.9	1.2
Karjalan Pyhäjärvi	2.2	5.5	13.3	0.8	0.4	1.3	10.4	1.8	0.2	8.3	0.5	0.5	0.8	12.8	4.9	0.9	1.5	11.6	9.7	6.4	4.6	3.1	1.0	1.2	0.9
Ylinen	2.3	6.7	12.7	0.6	0.4	0.8	20.9	0.5	0.0	13.5	0.9	0.9	1.3	6.1	4.9	0.8	0.8	7.4	9.2	5.5	5.6	2.2	0.8	0.4	0.7
Kermajärvi	2.3	7.1	10.6	0.7	0.4	0.9	19.0	0.3	0.1	7.4	0.4	0.6	1.1	7.8	3.7	0.7	1.1	9.1	11.5	8.1	7.0	2.4	0.9	1.1	0.8
Koitere	6.5	5.4	11.6	0.8	0.4	1.2	21.1	0.4	0.2	4.0	0.4	0.6	0.9	11.4	4.2	1.6	1.2	9.8	12.5	5.8	3.9	2.4	1.3	1.6	0.9
Nuorajärvi	6.1	6.5	10.0	1.1	0.6	4.0	20.9	0.5	0.3	3.4	0.0	0.6	0.7	14.4	1.6	1.1	1.7	10.0	11.0	7.8	3.1	1.5	0.7	2.3	0.7
Hattujärvi	7.4	7.3	5.1	0.6	0.3	6.8	26.8	0.5	0.1	2.6	0.2	1.2	1.1	7.0	3.0	1.5	0.7	8.8	8.5	8.0	8.7	1.9	0.8	1.6	0.5
Harkkojärvi	8.2	5.7	4.1	1.0	0.5	2.0	24.6	0.5	0.2	4.0	0.2	0.6	0.7	7.7	3.3	0.9	1.7	17.2	9.7	6.7	3.6	3.3	0.9	1.8	0.6
Ätäskö	6.4	5.3	12.5	1.6	0.6	1.1	11.4	1.8	0.3	7.8	1.7	0.8	1.2	17.2	3.4	0.9	2.1	9.6	6.3	6.9	4.3	2.1	0.9	1.8	0.7
Mekrijärvi	9.5	5.1	11.7	1.1	0.5	4.7	22.9	0.6	0.2	3.1	0.0	0.8	0.8	11.6	3.2	0.9	1.5	10.7	8.4	5.9	3.1	2.4	0.6	2.0	0.8

Table S4. Correlation coefficient, P-value and 95% confidence intervals between the measured fatty acid concentrations and calculated Chl-*a* weighed values for seston samples from boreal and temperate regions. Data are from Strandberg et al. (2020, 2022). The Chl-*a* weighed values and concentrations of EPA and DHA were strongly correlated in both regions. The measured concentrations and estimated Chl-*a* weighed values for 16:0 and 18:0 showed weaker, albeit significant, correlation and/or greater uncertainty. 16:0 and 18:0 are ubiquitous fatty acids; thus, the weaker correlation was most likely due to contribution from non-Chl-*a* containing sources, such as detritus.

Region	FA	r	P	95% Confidence interval
Temperate	14:0	0.96	< 0.001	0.645 - 0.985
	16:0	0.51	0.001	0.151 - 0.826
	18:0	0.61	< 0.001	0.334 - 0.925
	16:1n-7	0.93	< 0.001	0.550 - 0.977
	18:1n-9	0.84	< 0.001	0.654 - 0.923
	18:1n-7	0.80	< 0.001	0.545 - 0.933
	16:4n-3	0.97	< 0.001	0.791 - 0.988
	18:2n-6	0.90	< 0.001	0.539 - 0.962
	18:3n-6	0.88	< 0.001	0.586 - 0.977
	18:3n-3	0.88	< 0.001	0.576 - 0.977
	18:4n-3	0.85	< 0.001	0.750 - 0.998
	20:4n-6	0.94	< 0.001	0.676 - 0.986
	20:5n-3	0.96	< 0.001	0.823 - 0.992
	22:5n-6	0.87	< 0.001	0.811 - 0.981
	22:6n-3	0.98	< 0.001	0.900 - 0.991
Boreal	14:0	0.90	< 0.001	0.684 - 0.993
	16:0	0.96	< 0.001	0.071 - 0.999
	18:0	0.65	0.043	0.045 - 0.959
	16:1n-7	0.85	0.002	0.610 - 0.996
	18:1n-9	0.91	< 0.001	0.790 - 0.989
	18:1n-7	0.94	< 0.001	0.877 - 0.986
	16:4n-3	0.87	0.001	0.558 - 0.999
	18:2n-6	0.92	< 0.001	0.755 - 0.993
	18:3n-6	0.90	< 0.001	0.691 - 0.993
	18:3n-3	0.93	< 0.001	0.824 - 0.991
	18:4n-3	0.93	< 0.001	0.857 -0.990
	20:4n-6	0.91	< 0.001	0.740 - 0.999
	20:5n-3	0.89	< 0.001	0.774 - 0.995
	22:5n-6	0.90	< 0.001	0.736 - 0.979
	22:6n-3	0.96	< 0.001	0.655 - 0.995

Table S5. Mean fatty acids proportion (weight % of total fatty acids) in **filter-feeding cladocerans** from the boreal (North Karelia, Finland) and temperate (Ontario, Canada) lakes. The fatty acid data include the major fatty acids, which account for 97.7 – 99.7 % of total fatty acids.

LAKE	14:0	i15:0	ai15:0	15:0	16:0	i17:0	17:0	18:0	20:0	16:1n-9	16:1n-7	18:1n-9	18:1n-7	18:2n-6	18:3n-3	18:4n-3	20:4n-6	20:4n-3	20:5n-3	22:5n-6	22:5n-3	22:6n-3	24:1n-9
Bass	5.2	0.9	0.3	1.3	21.3	0.5	1.7	6.0	0.3	0.9	4.6	6.3	2.8	5.0	8.0	5.2	3.9	0.6	10.3	3.2	0.5	9.5	0.7
Bella	6.7	1.2	0.6	1.5	13.2	0.6	1.4	5.4	0.4	1.3	4.2	5.6	4.7	5.4	6.5	4.8	13.3	0.5	15.7	1.4	0.2	4.3	0.6
Couchiching	7.7	1.6	0.5	1.5	17.0	1.0	1.3	6.1	0.3	1.1	5.0	4.6	2.9	6.6	5.7	5.4	7.4	0.6	12.4	2.3	0.5	7.3	0.4
Crystal	4.0	1.0	0.3	1.2	18.9	0.8	1.5	5.9	0.4	1.1	3.9	7.6	3.3	4.9	8.6	6.0	3.6	0.7	11.6	3.0	0.4	9.9	0.8
Davis	4.6	1.0	0.4	1.1	20.1	1.0	1.6	5.7	0.7	1.6	4.5	8.7	5.8	6.4	7.2	4.0	5.9	1.3	7.1	2.7	0.9	5.8	0.6
Depensiers	7.2	0.9	0.5	2.1	16.2	0.5	1.5	6.5	0.3	0.9	3.0	3.9	2.2	5.4	7.3	6.6	4.6	2.1	8.9	3.2	0.8	12.9	0.4
Devil's	5.8	0.9	0.4	1.2	18.6	0.4	0.9	5.3	0.4	1.4	3.1	8.4	3.7	6.2	8.4	5.9	6.3	0.7	8.4	3.6	0.6	7.5	0.7
Four Mile	4.7	0.9	0.3	1.2	18.1	0.5	1.7	5.0	0.5	1.1	3.9	11.4	3.7	5.7	9.3	5.9	5.4	0.4	13.1	1.5	0.2	4.2	0.7
Fox	4.9	1.0	0.4	1.3	16.3	0.6	1.2	5.3	0.4	1.4	3.6	8.7	4.7	6.1	9.9	6.3	6.2	0.7	10.4	2.7	0.4	6.0	0.6
Head	9.1	0.8	0.3	1.1	17.0	0.5	1.1	6.9	0.4	0.8	3.2	4.2	1.4	7.4	5.9	7.8	5.0	1.5	8.5	3.8	0.7	10.9	0.4
Kashagawigamog	5.7	1.0	0.4	1.3	18.7	0.9	1.5	6.0	0.4	1.1	4.4	5.3	4.1	6.0	7.4	5.2	6.6	0.8	9.3	3.2	0.6	8.6	0.5
Koshlong	8.8	1.3	0.4	1.5	14.6	0.7	1.3	5.6	0.3	1.3	3.4	6.9	4.0	5.2	6.1	4.2	12.6	0.5	14.6	1.7	0.2	4.0	0.1
Loom	3.9	1.1	0.5	2.1	17.2	0.6	1.5	4.4	0.3	1.0	4.4	7.2	4.6	5.0	14.5	7.8	3.6	0.5	12.6	1.5	0.2	4.3	0.5
MacLean	7.0	2.7	0.7	2.2	19.6	1.8	2.1	8.3	0.3	1.1	4.4	8.6	2.7	7.0	5.3	2.9	4.2	1.2	5.6	1.8	0.6	7.4	0.2
Maple	6.0	1.1	0.4	1.3	17.1	0.7	1.3	5.6	0.4	1.1	3.9	5.2	3.3	5.0	8.0	6.3	5.0	1.2	9.7	3.3	0.9	11.3	0.7
Mary	5.4	1.3	0.5	1.3	18.2	0.9	1.5	6.7	0.3	1.2	4.6	5.2	4.0	4.8	6.0	4.0	5.7	0.9	10.7	3.6	0.5	10.9	0.9
Mink	5.3	1.1	0.7	2.7	15.8	0.6	1.5	6.0	0.3	1.6	6.0	6.0	5.2	4.7	4.9	7.1	7.0	0.8	11.3	3.1	0.4	6.6	0.4
Morrisson	5.1	0.9	0.3	1.9	18.4	0.6	1.5	7.0	0.4	1.0	3.8	6.7	4.0	5.1	7.5	4.7	6.7	0.8	9.3	3.4	0.8	8.6	0.5
Oxbow	7.8	1.0	0.5	1.2	13.2	0.7	1.1	4.6	0.5	1.4	4.0	4.6	3.5	4.5	7.7	10.3	6.9	0.6	12.8	3.2	0.4	8.0	0.5
Paint	7.0	0.9	0.4	1.2	15.8	0.6	1.1	4.6	0.4	1.1	3.1	5.2	2.6	5.1	8.4	6.8	6.2	0.9	13.2	2.8	0.6	10.4	0.8
Raven	8.7	1.1	0.5	1.4	14.3	0.6	1.2	5.5	0.4	1.4	3.2	6.3	4.0	4.7	6.0	5.1	11.7	0.8	12.9	2.8	0.5	5.8	0.2
Ril	8.1	1.1	0.5	2.6	17.4	0.6	1.7	5.3	0.4	1.0	3.8	5.4	2.6	4.4	8.0	8.6	4.5	0.9	8.2	4.1	0.7	8.3	0.4
Sparrow	5.2	1.1	0.3	1.0	21.6	0.6	1.4	6.4	0.3	1.0	5.1	6.4	3.9	6.1	7.2	4.0	4.7	0.6	9.2	3.1	0.6	9.0	0.6
Tea	4.3	0.8	0.2	1.2	17.9	0.5	1.5	5.8	0.3	0.9	4.0	9.2	4.5	6.1	7.4	4.8	7.1	0.6	11.3	3.0	0.4	7.2	0.4
Twelve Mile	8.2	1.1	0.4	1.2	14.6	0.6	0.9	5.2	0.4	1.1	3.5	5.2	3.0	4.9	6.6	7.0	8.7	0.6	14.7	2.5	0.4	7.8	1.0
Wasi	4.2	1.5	0.3	1.4	18.7	0.6	1.6	4.3	0.2	0.7	2.9	3.7	2.3	3.9	14.1	10.8	2.5	1.0	11.5	3.0	0.6	8.4	0.6
Harkkojärvi	4.4	3.1	0.9	1.4	23.1	0.8	1.6	6.9	0.1	1.3	10.9	12.1	7.5	2.7	4.4	2.1	5.4	0.2	8.5	0.0	0.0	0.3	0.0
Hattujärvi	6.1	2.6	0.9	1.5	22.8	0.7	1.0	7.5	0.2	1.0	9.0	9.2	9.6	2.9	5.2	3.8	4.6	0.2	9.6	0.0	0.0	0.5	0.0
Karjalan Pyhäjärvi	7.3	1.0	0.3	0.9	23.2	0.3	1.1	4.1	0.1	0.7	6.3	9.9	4.6	4.9	9.2	8.8	4.9	0.1	11.6	0.0	0.0	0.4	0.0
Kermajärvi	6.4	0.7	0.2	1.4	21.5	0.4	1.0	4.1	0.1	0.3	3.6	7.8	4.5	3.7	4.8	3.1	4.6	0.7	14.1	1.6	0.2	13.4	1.0
Koitere	12.0	1.6	0.5	1.2	26.0	0.5	0.6	6.0	0.1	0.7	8.9	7.5	4.4	3.7	6.0	5.6	4.7	0.2	8.0	0.2	0.0	1.0	0.0
Kuorinka	11.6	1.4	0.6	1.1	27.0	0.8	0.9	7.3	0.1	0.7	5.1	8.3	4.1	5.1	3.6	4.4	5.5	0.2	10.7	0.0	0.0	0.7	0.0
Mekrijärvi	11.5	2.6	0.8	1.9	27.7	0.7	1.5	6.3	0.1	1.1	13.5	8.2	4.5	3.5	4.8	2.6	3.0	0.1	4.4	0.1	0.0	0.3	0.0
Nuorajärvi	7.5	2.8	0.7	1.9	26.8	0.7	1.4	7.0	0.2	1.0	12.8	8.7	6.5	2.3	4.9	2.7	4.1	0.1	6.7	0.1	0.0	0.2	0.0
Ylinen	10.9	1.3	0.5	0.9	25.9	0.5	1.0	5.4	0.1	0.6	4.3	9.6	4.5	4.5	6.6	5.7	4.5	0.3	10.3	0.2	0.1	1.7	0.0
Ätäskö	7.6	2.7	0.5	1.2	21.8	0.8	1.6	4.6	0.1	0.7	14.8	5.5	5.7	2.5	5.8	3.5	6.1	0.1	12.8	0.2	0.0	0.5	0.0

Table S6. Mean fatty acids proportion (weight % of total fatty acids) in **predatory cladocerans** from the boreal lakes (North Karelia, Finland). Predatory cladocerans were not analyzed from the temperate lakes (Ontario, Canada). The fatty acid data include the major fatty acids, which account for 99.7 – 99.9 % of total fatty acids.

LAKE	14:0	i15:0	ai15:0	15:0	i16:0	16:0	i17:0	17:0	18:0	20:0	16:1n-9	16:1n-7	18:1n-9	18:1n-7	18:2n-6	18:3n-3	18:4n-3	20:4n-6	20:4n-3	20:5n-3	22:6n-3
Harkkojärvi	6.1	1.5	0.3	2.0	0.5	26.6	0.5	2.9	7.9	0.0	1.4	7.1	16.6	4.4	3.6	3.9	0.5	5.1	0.2	8.4	0.2
Hattujärvi	5.8	1.1	0.3	1.4	0.2	31.8	0.4	1.9	12.1	0.1	0.9	8.1	13.1	5.1	1.9	3.3	0.5	3.5	0.1	8.3	0.0
Karjalan Pyhäjärvi	6.9	0.7	0.2	1.4	0.2	32.4	0.3	2.6	11.7	0.1	1.5	4.4	18.6	3.0	1.6	3.1	0.4	3.8	0.1	6.7	0.3
Kuorinka	6.9	0.8	0.3	1.4	0.3	35.7	0.4	1.5	15.8	0.2	0.9	3.1	12.1	3.3	3.3	3.1	0.4	3.9	0.1	6.3	0.1
Mekrijärvi	8.6	1.0	0.3	2.2	0.2	31.1	0.3	2.6	12.0	0.1	1.0	5.8	10.6	2.4	3.5	3.8	0.7	4.4	0.4	8.4	0.2
Nuorajärvi	8.0	1.1	0.2	3.2	0.2	34.7	0.4	3.0	13.5	0.1	0.9	5.6	12.4	3.0	2.2	2.9	0.2	3.0	0.1	5.1	0.0
Ylinen	6.9	0.9	0.2	1.1	0.3	31.3	0.5	2.1	12.3	0.2	1.4	3.1	16.5	3.4	2.7	3.8	0.6	5.0	0.1	7.1	0.3

Table S7. Mean fatty acids proportion (weight % of total fatty acids) in **copepods from the temperate (Ontario, Canada) lakes**. Copepods from the boreal lakes (North Karelia, Finland) are presented in Tables S8 and S9. The fatty acid data include the major fatty acids, which account for 98.8 – 99.8 % of total fatty acids.

Lake	14:0	i15:0	ai15:0	15:0	i16:0	16:0	i17:0	17:0	18:0	20:0	16:1n-9	16:1n-7	18:1n-9	18:1n-7	18:2n-6	18:3n-3	18:4n-3	20:4n-6	20:3n-3	20:4n-3	20:5n-3	22:5n-6	22:5n-3	22:6n-3	24:1n-9
Bass	4.2	0.7	0.2	1.2	0.2	20.1	0.4	1.8	6.8	0.3	0.6	3.3	5.0	2.4	4.1	7.5	4.7	3.6	0.2	0.7	11.0	4.6	0.7	14.0	0.9
Bella	6.4	1.1	0.5	1.2	0.3	16.7	0.7	1.3	4.4	0.6	1.2	3.9	5.1	2.1	4.6	8.8	7.3	2.9	0.2	0.6	10.8	3.7	0.5	13.2	1.1
Brandy	4.5	1.6	0.5	2.0	0.8	17.6	1.0	1.9	6.0	0.4	1.0	3.4	5.2	2.9	5.8	10.8	6.6	4.6	0.6	1.2	8.6	3.2	1.0	8.3	0.4
Couchiching	7.2	1.5	0.5	1.5	0.3	16.8	1.0	1.4	6.0	0.3	1.0	4.8	4.4	2.8	5.8	5.3	5.0	6.9	0.2	0.7	11.9	3.0	0.7	10.3	0.5
Crystal	5.8	1.1	0.4	1.1	0.3	19.4	0.9	1.6	6.1	0.4	0.9	2.6	4.7	2.1	3.9	6.5	4.6	3.4	0.2	0.7	10.7	4.7	0.5	15.3	1.8
Davis	5.1	1.0	0.4	1.1	0.4	20.0	1.1	1.6	6.4	0.7	1.5	4.2	5.9	4.6	5.3	6.0	3.6	4.9	0.6	1.6	6.6	4.6	1.3	10.3	0.9
Depensiers	7.1	1.0	0.5	2.1	0.3	16.4	0.5	1.6	6.7	0.3	1.0	3.1	3.9	2.2	5.4	7.2	6.3	4.7	0.3	2.3	8.7	3.2	0.8	12.8	0.4
Devil's	5.6	0.9	0.4	1.1	0.3	19.4	0.5	1.0	5.7	0.5	1.3	3.1	6.7	2.9	5.3	7.1	5.1	4.8	0.3	0.9	8.1	5.2	0.7	11.5	1.1
Four Mile	6.0	1.0	0.4	0.9	0.3	18.8	0.7	1.5	6.2	0.5	0.7	2.4	5.5	1.8	4.7	5.8	4.5	4.2	0.1	0.5	10.5	4.9	0.5	16.1	1.3
Fox	6.5	1.2	0.5	1.2	0.4	18.1	0.6	1.1	4.4	0.6	1.2	3.0	4.8	2.6	4.7	10.7	8.6	3.3	0.2	0.8	8.4	4.9	0.6	10.3	0.9
Head	10.6	0.9	0.3	1.2	0.3	16.0	0.6	1.2	6.4	0.5	0.7	2.6	3.6	1.1	6.8	6.0	8.1	4.3	0.2	1.6	7.7	4.6	0.9	13.1	0.4
Kash	5.6	1.0	0.4	1.3	0.3	18.4	0.8	1.3	5.7	0.4	1.2	4.4	4.9	3.8	5.9	7.5	5.6	6.3	0.3	0.8	9.5	3.4	0.7	9.6	0.8
Kosh	5.2	0.9	0.3	1.1	0.2	16.0	0.7	1.3	7.8	0.3	1.0	2.3	4.6	2.8	4.0	4.7	3.7	6.0	0.2	0.8	13.4	5.5	0.6	15.5	1.0
Loom	5.7	1.1	0.4	1.2	0.4	19.7	1.0	1.6	5.9	0.3	1.2	4.0	5.0	3.9	5.0	7.6	5.5	4.5	0.2	0.5	9.9	3.7	0.4	10.1	1.0
Loon	9.1	1.2	0.5	1.2	0.3	18.1	0.6	0.8	4.2	0.4	0.7	3.0	3.0	2.0	4.4	8.6	10.6	3.5	0.1	0.8	9.3	3.8	0.6	12.3	0.7
MacLean	6.7	2.1	0.6	2.0	1.2	20.0	1.6	2.0	8.4	0.4	1.0	3.9	7.7	2.3	7.6	5.7	3.4	3.6	0.3	1.3	5.5	2.0	0.7	9.0	0.2
Maple	5.6	1.0	0.4	1.1	0.3	17.0	0.8	1.5	6.2	0.5	1.1	3.5	4.5	3.1	4.8	7.3	5.3	4.8	0.4	1.2	9.6	4.0	1.1	13.9	0.8
Mary	4.7	1.0	0.3	1.0	0.4	17.8	0.7	1.5	6.0	0.4	1.0	3.6	4.2	3.1	4.1	5.6	3.8	4.7	0.3	1.0	10.8	5.3	0.7	16.4	1.5
Menominee	7.7	1.0	0.4	2.5	0.3	16.9	0.5	1.4	5.6	0.3	0.8	3.5	4.1	2.2	4.1	6.3	7.3	5.0	0.2	0.8	7.9	6.3	0.6	13.1	0.6
Mink	6.3	0.7	0.5	3.1	0.3	16.3	0.4	1.5	6.0	0.3	1.0	3.6	4.6	2.9	4.3	5.2	7.0	5.4	0.2	0.9	9.9	5.5	0.5	12.2	0.6
Morrisson	4.7	0.9	0.3	2.0	0.2	18.6	0.5	1.6	7.5	0.4	1.0	3.3	5.6	3.3	4.8	6.8	4.3	6.2	0.3	0.9	9.1	4.2	1.0	11.3	0.6
Oxbow	8.4	0.9	0.4	1.0	0.4	14.1	0.5	0.9	4.1	0.6	1.3	3.5	3.7	2.0	4.5	8.2	12.3	3.6	0.2	0.7	10.4	4.6	0.6	11.8	0.9
Paint	6.3	0.8	0.3	1.1	0.3	16.0	0.5	1.1	4.6	0.5	0.5	2.5	4.8	1.9	4.8	9.4	7.7	3.9	0.3	1.0	11.9	3.9	0.8	13.8	0.8
Raven	7.5	0.8	0.4	1.1	0.3	15.0	0.5	1.2	6.1	0.4	1.3	2.7	4.7	2.6	4.4	6.9	6.2	6.5	0.3	1.2	9.9	5.8	0.9	12.1	0.6
Ril	7.2	0.9	0.4	3.0	0.3	17.6	0.6	1.8	5.9	0.4	0.8	2.9	3.8	1.8	4.0	7.3	6.8	4.2	0.3	1.1	7.8	5.6	1.0	13.0	0.6
Sparrow	4.8	0.9	0.2	0.9	0.3	21.1	0.5	1.5	6.8	0.3	0.8	3.8	5.4	3.2	5.5	6.4	3.5	4.6	0.2	0.6	9.7	4.3	0.7	13.0	0.9
Tea	5.4	0.9	0.3	1.1	0.2	19.0	0.5	1.4	6.0	0.3	0.7	3.3	5.7	2.5	5.1	6.5	5.3	5.0	0.2	0.7	10.2	5.3	0.5	13.0	0.8
Twelve Mile	7.9	1.1	0.4	1.0	0.2	16.4	0.7	1.2	5.2	0.5	1.0	3.5	4.4	2.3	4.2	7.1	7.1	4.0	0.2	0.8	10.6	4.5	0.6	14.0	0.7
Wasi	3.7	1.2	0.2	1.0	0.2	18.6	0.5	1.8	4.4	0.2	0.5	1.5	2.5	1.9	3.3	12.3	9.1	2.1	0.4	1.1	12.8	4.5	0.8	14.1	0.8

Table S8. Mean fatty acids proportion (weight % of total fatty acids) in **copepods (Cyclopoida, *Eudiaptomus*, *Heterocope*) from the boreal lakes** (North Karelia, Finland). The fatty acid data include the major fatty acids, which account for 98.6 – 100 % of total fatty acids.

Taxa/Lake	14:0	i15:0	ai15:0	15:0	i16:0	16:0	i17:0	17:0	18:0	20:0	16:1n-9	16:1n-7	18:1n-9	18:1n-7	18:2n-6	18:3n-3	18:4n-3	20:4n-6	20:4n-3	20:5n-3	22:5n-6	22:5n-3	22:6n-3	24:1n-9
**Cyclopoida**																								
Karjalan Pyhäjärvi	10.5	1.1	0.5	0.7	0.2	20.1	0.3	0.8	5.6	0.4	0.2	11.7	3.3	2.2	3.6	5.8	7.3	1.2	2.4	6.9	2.0	2.2	9.2	0.7
Kermajärvi	6.2	1.4	0.4	1.5	0.4	24.5	0.5	0.0	6.4	0.4	0.2	2.5	3.9	2.9	3.9	6.5	3.1	2.1	1.0	9.9	2.8	2.1	14.1	1.7
Koitere	6.5	1.9	0.2	2.0	0.3	29.3	0.6	1.9	12.0	0.5	0.1	2.2	2.0	2.1	2.0	4.3	1.8	2.5	0.4	6.4	3.7	0.9	15.0	0.7
Ylinen	7.9	1.8	0.4	1.5	0.5	24.8	0.8	1.5	8.3	0.5	0.3	2.3	5.0	2.4	4.5	6.8	4.1	2.0	1.7	6.5	2.5	1.5	10.7	0.4
Eudiaptomus																								
Harkkojärvi	5.4	1.1	0.2	1.6	0.4	24.3	0.5	1.4	6.1	0.3	0.2	2.2	1.9	1.6	3.0	7.5	2.6	3.6	0.2	11.7	5.3	0.3	17.2	1.3
Hattujärvi	6.7	0.7	0.6	2.5	0.8	28.8	0.4	1.7	9.3	0.7	3.8	3.3	6.0	1.5	2.3	4.5	2.0	2.5	0.1	7.7	2.5	0.2	10.4	0.7
Karjalan Pyhäjärvi	9.5	1.0	0.3	0.7	0.2	31.0	0.3	1.0	4.9	0.3	0.2	1.4	3.4	1.2	2.6	7.2	4.6	1.6	0.3	8.3	3.9	0.2	14.6	1.2
Kermajärvi	7.8	0.6	0.2	0.8	0.3	26.0	0.3	0.9	6.0	0.3	0.8	1.4	4.6	1.4	3.2	5.6	4.5	2.1	0.2	9.8	3.0	0.5	17.9	1.7
Koitere	7.5	2.0	0.3	1.6	0.5	26.8	0.6	1.1	5.8	0.3	0.4	3.0	2.2	1.8	2.6	8.0	4.1	3.1	0.2	9.5	5.0	0.3	12.4	0.9
Kuorinka	7.3	0.5	0.2	0.4	0.5	28.7	0.3	0.7	9.3	0.1	0.1	1.3	4.5	1.2	2.9	2.0	1.2	3.1	0.1	7.2	3.2	0.0	23.1	2.0
Mekrijärvi	6.3	0.6	0.2	1.5	0.2	30.7	0.3	1.8	9.0	0.1	0.1	2.7	3.4	1.2	2.6	3.3	0.8	3.5	0.1	8.9	3.8	0.0	17.5	1.4
Nuorajärvi	5.2	0.5	0.1	1.3	0.5	38.0	0.3	1.5	13.0	0.1	0.1	1.7	3.0	1.3	1.7	2.2	0.2	2.7	0.0	6.1	3.5	0.0	15.7	1.4
Ylinen	7.7	0.0	0.2	0.4	0.2	31.1	0.3	0.9	7.8	0.2	0.0	0.8	4.1	1.1	3.0	5.3	2.7	1.7	0.0	7.4	3.2	0.0	20.0	1.9
Ätäskö	5.4	1.3	0.2	0.8	0.3	25.4	0.6	1.5	6.3	0.2	0.1	6.1	1.5	2.0	1.8	5.4	2.7	3.7	0.2	13.1	4.2	0.0	16.5	0.9
Heterocope																								
Harkkojärvi	6.5	1.1	0.2	1.9	0.5	28.1	0.6	1.8	9.7	0.1	0.1	1.9	1.7	1.9	1.9	3.3	0.6	3.0	0.4	11.2	4.1	0.6	16.9	1.5
Hattujärvi	6.3	0.8	0.1	1.9	0.1	31.1	0.2	0.9	11.8	0.1	0.1	9.0	2.1	3.0	1.6	3.1	1.1	1.8	0.2	8.4	1.5	1.6	11.5	1.3
Karjalan Pyhäjärvi	8.6	1.5	0.4	1.0	0.5	31.6	0.6	1.2	10.9	0.3	0.1	2.7	2.5	2.0	2.3	4.2	3.2	2.3	0.1	8.3	2.6	0.3	10.8	1.3
Kermajärvi	5.4	0.4	0.1	0.5	0.2	19.1	0.4	0.9	5.3	0.1	0.1	1.1	2.7	2.4	1.7	2.5	1.0	3.4	0.2	15.5	2.8	0.4	31.6	1.8
Koitere	6.6	0.7	0.2	1.2	0.2	22.0	0.3	0.9	7.3	0.1	0.1	2.1	2.3	2.5	1.9	4.0	1.7	3.1	0.3	14.5	3.1	0.5	23.0	1.1
Nuorajärvi	6.0	1.1	0.2	1.5	0.3	22.9	0.6	1.8	8.7	0.1	1.0	2.1	2.1	2.5	2.2	3.8	0.8	3.2	0.4	13.5	4.4	0.5	18.6	1.4
Ylinen	10.9	1.0	0.3	0.7	0.6	37.9	0.6	0.9	9.9	0.2	0.0	0.4	3.1	1.1	1.2	1.3	0.4	1.5	0.0	5.7	2.8	0.3	16.8	2.1

Table S9. Mean fatty acids proportion (weight % of total fatty acids) in ***Limnocalanus macrurus*** from the boreal lakes (North Karelia, Finland). The fatty acid data include the major fatty acids, which account for 99.0 – 99.7 % of total fatty acids.

Lake	14:0	i15:0	15:0	i16:0	16:0	i17:0	17:0	18:0	16:1n-7	18:1 n-9	18:1 n-7	18:2 n-6	18:3 n-3	18:4 n-3	20:4 n-6	20:3 n-3	20:4 n-3	20:5 n-3	22:5 n-6	22:5 n-3	22:6 n-3	24:1 n-9	24:4 n-3	24:5 n-3	24:6 n-3
Karjalan Pyhäjärvi	3.8	0.7	0.0	0.0	24.3	0.2	0.0	7.8	2.8	5.9	3.4	4.6	6.5	3.2	2.3	3.3	3.8	7.1	3.7	2.3	9.8	1.3	0.6	1.2	0.5
Kermajärvi	2.3	0.2	4.6	1.1	9.7	0.1	0.1	1.3	2.6	5.9	3.4	5.9	9.6	3.6	2.9	3.4	3.0	13.7	3.6	1.9	17.2	1.4	0.4	0.6	0.7
Kuorinka	6.7	0.4	2.3	0.4	29.8	0.2	0.2	4.2	2.2	4.0	3.4	3.1	2.0	0.8	2.7	0.4	0.9	10.1	2.5	0.3	21.2	1.2	0.7	0.0	0.0
Ylinen	1.6	0.2	4.8	1.1	7.9	0.1	0.1	0.9	1.5	4.8	2.2	5.0	9.0	2.7	2.7	6.2	3.4	15.8	4.9	4.1	13.5	1.4	1.1	2.5	1.7

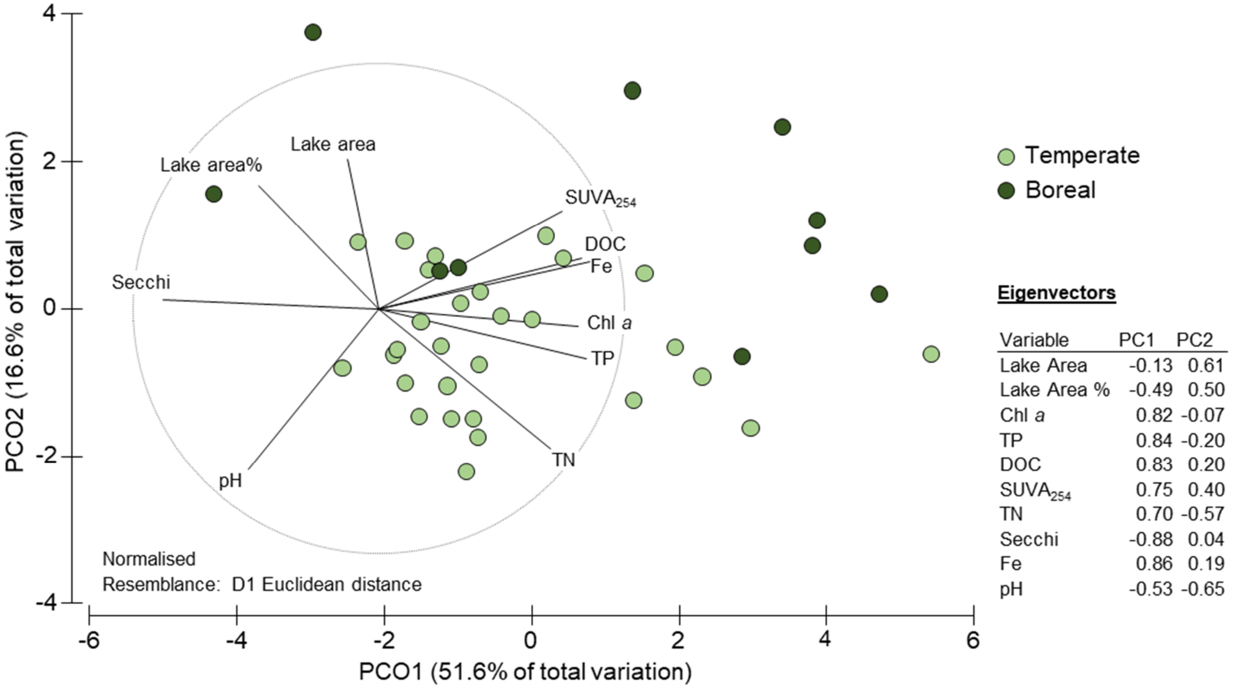

Eigenvectors

Variable	PC1	PC2
Lake Area	-0.13	0.61
Lake Area %	-0.49	0.50
Chl *a*	0.82	-0.07
TP	0.84	-0.20
DOC	0.83	0.20
SUVA$_{254}$	0.75	0.40
TN	0.70	-0.57
Secchi	-0.88	0.04
Fe	0.86	0.19
pH	-0.53	-0.65

Figure S1. Principal component analysis of selected environmental factors for temperate (n=29) and boreal (n=10) lakes.

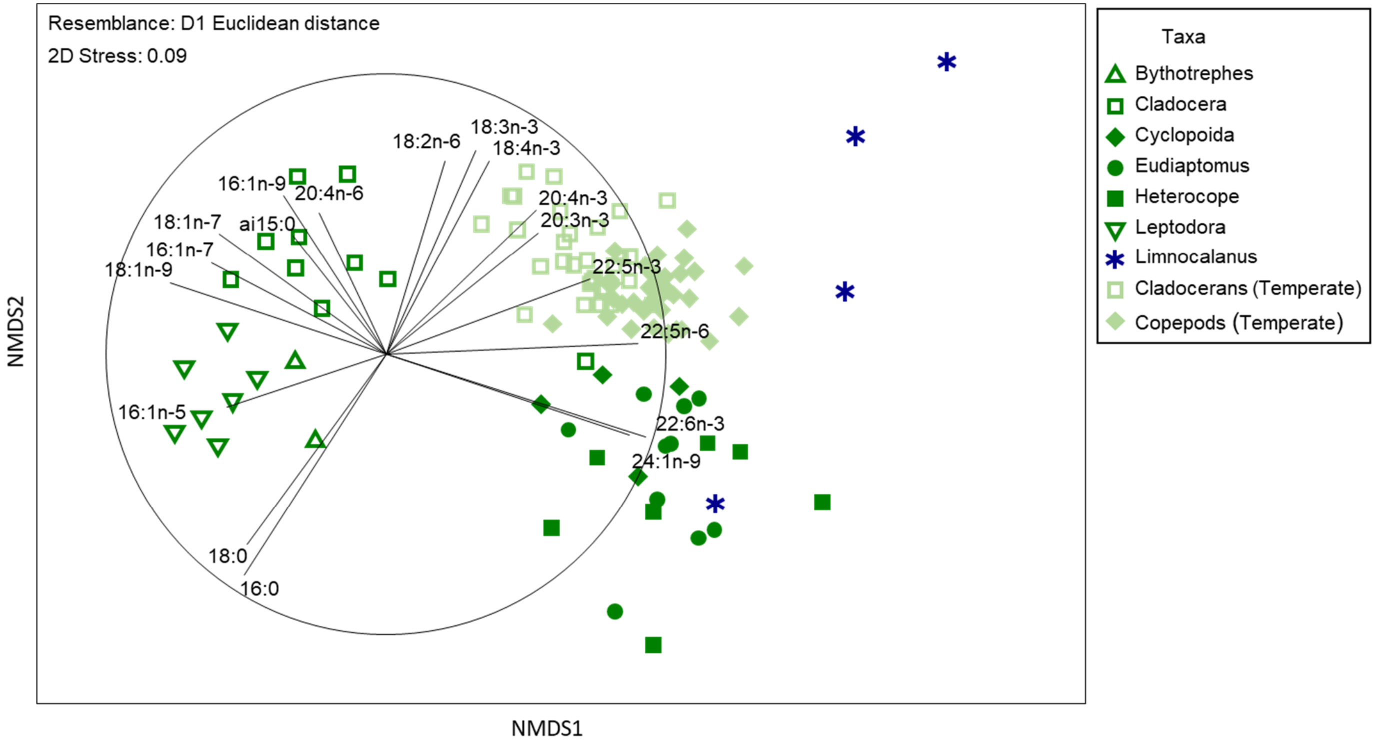

Figure S2. Non-metric multidimensional plot based on zooplankton fatty acid profiles (w%). Light green marks temperate zooplankton and dark green boreal zooplankton (see figure legend for details). *Limnocalanus macrurus* from oligotrophic clearwater boreal lakes is marked with blue asterix. Fatty acids that correlate with the dimensions ($r > 0.5$) are also presented. The vectors indicate the direction and strength of the correlation.
